# Prognostic factors for patients with anal cancer treated with conformal radiotherapy—a systematic review

**DOI:** 10.1186/s12885-022-09729-4

**Published:** 2022-06-03

**Authors:** Stelios Theophanous, Robert Samuel, John Lilley, Ann Henry, David Sebag-Montefiore, Alexandra Gilbert, Ane L. Appelt

**Affiliations:** 1 Leeds Institute of Medical Research, University of Leeds, Beckett Street, Leeds, LS9 7TF UK; 2 Department of Medical Physics, Leeds Cancer Centre, St James’s University Hospitals, Beckett Street, Leeds, LS9 7TF UK

**Keywords:** Systematic review, Anal cancer, Squamous cell carcinoma, Conformal radiotherapy, IMRT, VMAT, Cancer outcomes, Survival outcomes, Prognostic factors

## Abstract

**Aims:**

Anal cancer is primarily treated using concurrent chemoradiotherapy (CRT), with conformal techniques such as intensity modulated radiotherapy (IMRT) and volumetric arc therapy (VMAT) now being the standard techniques utilised across the world. Despite this, there is still very limited consensus on prognostic factors for outcome following conformal CRT. This systematic review aims to evaluate the existing literature to identify prognostic factors for a variety of oncological outcomes in anal cancer, focusing on patients treated with curative intent using contemporary conformal radiotherapy techniques.

**Materials and methods:**

A literature search was conducted using Medline and Embase to identify studies reporting on prognostic factors for survival and cancer-related outcomes after conformal CRT for anal cancer. The prognostic factors which were identified as significant in univariable and multivariable analysis, along with their respective factor effects (where available) were extracted. Only factors reported as prognostic in more than one study were included in the final results.

**Results:**

The results from 19 studies were analysed. In both univariable and multivariable analysis, N stage, T stage, and sex were found to be the most prevalent and reliable clinical prognostic factors for the majority of outcomes explored. Only a few biomarkers have been identified as prognostic by more than one study – pre-treatment biopsy HPV load, as well as the presence of leukocytosis, neutrophilia and anaemia at baseline measurement. The results also highlight the lack of studies with large cohorts exploring the prognostic significance of imaging factors.

**Conclusion:**

Establishing a set of prognostic and potentially predictive factors for anal cancer outcomes can guide the risk stratification of patients, aiding the design of future clinical trials. Such trials will in turn provide us with greater insight into how to effectively treat this disease using a more personalised approach.

**Supplementary Information:**

The online version contains supplementary material available at 10.1186/s12885-022-09729-4.

## Background

First reported in 1974 by Nigro et al. [1] and established by two phase III trials [2, 3], concurrent chemoradiotherapy (CRT) is the current standard of care for localised anal squamous cell carcinoma (ASCC). The introduction of three-dimensional conformal radiotherapy (3D-CRT), intensity modulated radiotherapy (IMRT) and latterly volumetric arc therapy (VMAT) [4] has allowed for substantial reduction in dose to pelvic organs at risk (OAR) and associated toxicity, with far fewer unplanned treatment breaks as a result. The current UK standard for anal cancer comprises of IMRT/VMAT and concurrent chemotherapy with 5-fluorouracil (5-FU) or capecitabine and mitomycin C (MMC), with surgery reserved as salvage treatment [5].

Anal cancer is a rare cancer, and only a handful of late phase clinical trials have been conducted over the last four decades [2, 3, 6–9]. Other than the single arm phase II RTOG 0529 [10] trial, these trials were conducted prior to widespread adoption of conformal radiotherapy techniques, such as 3D-CRT or IMRT/VMAT. Similarly, much of the published literature on prognostic factors in anal cancer consists of retrospective series, often small cohorts [11, 12] or cohorts of patients treated with older techniques [13, 14]. No systematic review of studies identifying prognostic factors after treatment with conformal radiotherapy has previously been conducted.

Despite advances in radiotherapy planning and delivery, locoregional control remains challenging, and patients usually fail locoregionally before getting metastatic disease. A UK multi-centre retrospective review by Shakir et al. [15] analysed 385 anal cancer patients treated with contemporary radiotherapy techniques, and demonstrated a 85.6% three-year overall survival. Initial complete clinical response rates were high at 86.7%, but over time 24.4% of patients relapsed, with the majority of relapses (83.4%) being local.

Establishing risk factors for oncological outcomes, in particular locoregional control following conformal chemoradiotherapy, could help optimise future treatment strategies and aid in the design and analysis of new clinical trials [16]. A consensus on prognostic factors could inform research by determining specific patient risk groups and the development of personalised treatment approaches, tailored to individual patient characteristics [17], and/or the introduction of novel agent combinations. This systematic review evaluates the literature to identify prognostic factors for a variety of disease-related outcomes in anal cancer, focusing on patients treated with curative intent using conformal radiotherapy techniques and contemporary treatment schedules.

## Methods

A systematic review was undertaken according to PRISMA 2020 [18]. A comprehensive literature search was conducted using the Medline and Embase databases, to identify studies reporting on prognostic factors for survival and cancer-related outcomes after conformal chemoradiotherapy for anal cancer. The search terms included ‘radiotherapy’ AND ‘anal cancer’ AND ‘prognostic factor’, as well as related terms (see Appendix A for the full search strategies). Only studies published after 1^st^ January 2000 and up to and including 30^th^ June 2020 were considered. An initial scoping search showed that no studies conducted prior to 2000 had a majority of patients treated using conformal techniques.

Studies were included if they: (1) comprised of at least 70% of patients treated with solely conformal radiotherapy techniques (3D-defined targets on CT, beams conformed to targets e.g. using multileaf collimators, 3D dose calculation and dose distribution optimisation), (2) reported survival or disease-related outcomes and (3) examined prognostic factors for outcomes using univariable (UVA) or multivariable (MVA) analysis. Studies were excluded if (1) patients were treated with 2D radiotherapy techniques and/or fields based solely on bony landmarks, if (2) cohorts included less than 100 patients or (3) were derived from population-level databases, or if (4) treatment with palliative intent. The cut-off of 100 patients was chosen to ensure that the prognostic factors identified are generalisable and to decrease the likelihood of identifying spurious prognostic factors from studies that suffer from small sample size bias. All (5) meta-analysis studies, reviews, animal model studies, conference abstracts/letters and studies without English translation were also excluded.

Two independent reviewers (ST and RS) screened and reviewed all relevant articles. A third independent reviewer (AA) assisted in reconciling differences in cases of disagreement. One reviewer (ST) extracted and analysed data from all relevant articles, including: study location, publication year, study design, source of participants, participant selection criteria, number of patients included, treatment period, radiotherapy technique administered, radiotherapy schedule, chemotherapy regimen, follow-up procedure, core clinical/patient characteristics, outcomes reported/definitions, statistical analysis used, prognostic variables tested, prognostic variables identified as significant and corresponding effect estimates. An independent reviewer (RS) repeated the data extraction from a subset (20%) of all relevant articles to ensure that the data extraction process was reproducible. The methodological quality of all relevant articles was assessed independently by two reviewers (ST, RS) using the National Institutes of Health (NIH) Quality Assessment Tool for Case Series Studies [19]. Any disagreements were reviewed independently by a third reviewer (AA) to achieve consensus.

Reported outcomes and outcome definitions were extracted from each study and stratified into nine categories for further analysis. Disease activity and survival outcomes were firstly grouped according to the CORMAC review [20], which was used as the initial reporting framework for outcome stratification. Additional categories were inductively derived after the data extraction process.

For each study, factors analysed for their prognostic impact were extracted, whether they were shown to have a significant relationship with outcome, and the statistical method used for analysis. The factors were grouped into three broader categories: clinical factors, biomarkers and imaging factors. The total number of times a factor was tested in UVA for each of the nine outcomes was counted across all studies. Where factors tested were not reported explicitly, it was assumed that all reported patient characteristics were tested. Prognostic factors which were identified as significant in each study, along with their respective factor effect in the form of hazard ratios (HRs) were extracted (where available), and the proportion of times each factor was identified as prognostic for each outcome was calculated. Since the majority of studies did not report which factors were tested in MVA for each distinct outcome, the total number of times each factor was tested could not be counted. Therefore, only the prognostic factors and their respective factor effects were extracted. Only factors reported as prognostic in more than one study were included in the final results.

## Results

### Literature search

**Fig. 1:**
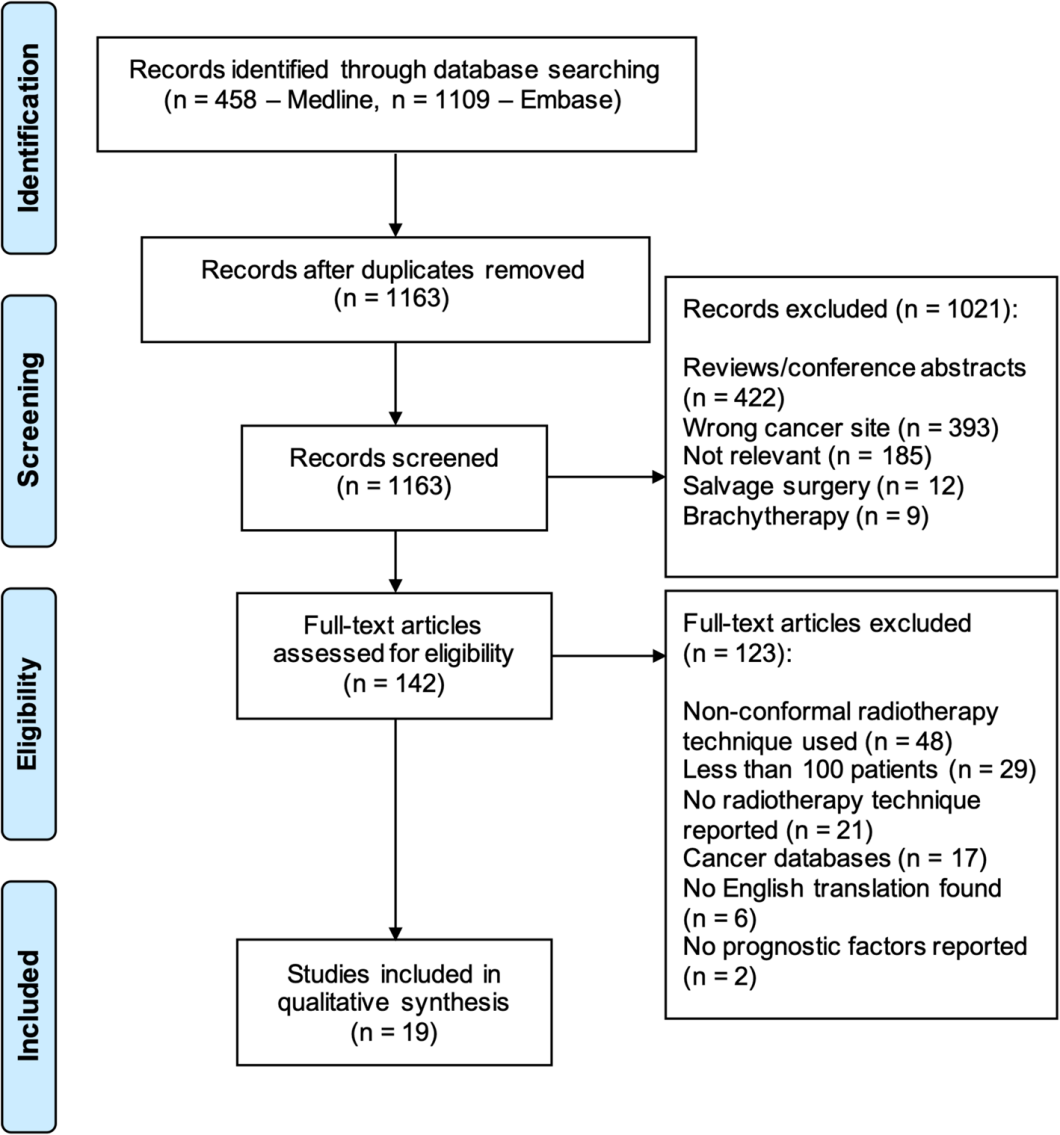
PRISMA flow diagram depicting the number of studies that were identified, included and excluded, and the reasons for exclusion

1567 studies published between 1^st^ January 2000 and 30^th^ June 2020 were identified, 404 of which were duplicates. Titles and abstracts of 1163 unique studies were screened. 1021 were excluded and the final 142 studies assessed for eligibility, of which 123 were excluded after reviewing the full text. 48 studies employed non-conformal radiotherapy techniques in more than 30% of patients. Other main factors for exclusion were sample size less than 100 (*n* = 29) and incomplete reporting on the radiotherapy technique (*n* = 21). Ultimately, 19 studies [15, 21–38] were included in this literature review (Fig. 1).

### Study characteristics

Included studies were retrospective case series (*n* = 19), either single institutional (*n* = 10) or multi-institutional (*n* = 9). Patients were treated between 1989–2018 with median follow-up range of 14.9–70.0 months. The most common radiotherapy techniques employed were a combination of 3D-CRT and IMRT/VMAT (*n* = 9), followed by IMRT only (*n* = 6). Dose ranged from 45 Gy/25 fractions to 63 Gy/35 fractions and chemotherapy regimens were mainly MMC and 5-FU based, with three studies including the option of cisplatin. Statistical techniques for UVA were log-rank tests (*n* = 12) and univariable Cox regression (*n* = 9), with four studies using both. Multivariable Cox regression was applied for MVA in all but one study, which used logistic regression instead. Regarding quality, 16 were deemed good and three deemed fair (Appendix B). A short follow-up (of less than 36 months, as used for the primary endpoint in the PLATO trial [17]) was a common issue in eight studies. Due to the lack of universal reporting of effect sizes for prognostic factors, it was not possible to carry out a meta-analysis on the data. Table 1 presents the main characteristics for all included studies (Appendix C presents a more detailed version including information on cancer subtype and location in the included cohorts, TNM staging version used and all predictors tested).

**Table 1:** Overview of study characteristics, including treatment techniques and regimens

#	**Study**	**Location**	**Number of patients**	**Years of treatment**	**Radiotherapy technique**	**Radiotherapy regimen**	**Chemotherapy regimen**	**Median follow-up (months)**	**Type of statistical analysis used**	**Quality**
1	Shakir et al. (2020) [15]	MC, EU	385	2013–2018	IMRT	50.4 Gy/28 fractions for T1/2N0, 53.2 Gy/28 fractions for T1/2 N + or T3/4Nany	MMC and Cap or 5-FU	24.0	UV Cox, MV Cox	Good
2	Martin et al. (2020) [21]	SC, EU	223	1996–2017	3D-CRT (58%) IMRT (42%)	50–50.4 Gy in 1.8–2 Gy/fraction, boost of 5.4–9 Gy	5-FU and MMC or Cisp	46.0	UV Cox, MV Cox	Good
3	de Bellefon et al. (2020) [22]	SC, EU	193	2005–2017	IMRT	45 Gy in 1.8 Gy/fraction, boost of 14.4–20 Gy (1.8–2 Gy/fraction)	5-FU and MMC	70.0	UV Cox, MV Cox	Good
4	Brown et al. (2019) [23]	SC, EU	189	2008–2016	2D/ 3D-CRT (79%) VMAT (21%)	49.6 Gy in 1.8 Gy/fraction	5-FU and MMC	35.1	MV logistic	Good
5	Rouard et al. (2019) [24]	MC, EU	165	2006–2016	IMRT	45–50 Gy in 1.8 or 2 Gy/fraction, boost of 15–20 Gy	5-FU and MMC	33.8	BV Cox, MV Cox	Good
6	Franco et al. (2018) [25]	MC, EU	161	NR	IMRT	50–50.4 Gy in 1.8–2 Gy/fraction	5-FU and MMC	27.0	Log-rank, UV Cox, MV Cox	Good
7	Call et al. (2016) [26]	MC, NA	152	NR	IMRT	51.25 Gy/28 fractions	5-FU and MMC (75% of patients)	26.8	Log-rank, MV Cox	Fair
8	Balermpas et al. (2017) [27]	MC, EU	150	NR	3D-CRT IMRT	53.4 Gy in 1.8–2 Gy/fraction	5-FU and MMC	40.0	Log-rank, MV Cox	Good
9	Rodel et al. (2018) [28]	MC, EU	140	NR	3D-CRT IMRT	53.4 Gy (range 46.8–64.8 Gy)	5-FU and MMC	40.0	Log-rank, MV Cox	Good
10	Schernberg et al. (2017) [29]	MC, EU	133	2000–2015	IMRT (77%) 3D-CRT (23%)	49.5 Gy/30 fractions (centre 1), 45 Gy/25 fractions (centre 2)	Cisp and 5-FU or Cap / MMC and 5-FU or Cap	37.4	Log-rank, MV Cox	Good
11	Martin et al. (2019) [30]	SC, EU	126	2004–2016	IMRT (65%) 3D-CRT (35%)	59.4 Gy in 1.8 or 2 Gy/fraction	5-FU and MMC	NR	Log-rank, UV Cox, MV Cox	Good
12	Oehler-Janne et al. (2008) [31]	MC, IN	121	1997–2006	3D-CRT	52 Gy-60 Gy depending on centre	5-FU and MMC or Cisp	36.0	Log-rank, UV Cox, MV Cox	Good
13	Susko et al. (2020) [32]	SC, NA	111	2005–2018	3D-CRT IMRT	55.8 Gy/30 fractions	5-FU and MMC	28.0	Log-rank, UV Cox, MV Cox	Good
14	Cardenas et al. (2017) [33]	SC, NA	110	2003–2013	IMRT (75%) 2D-CRT (25%)	50.4 Gy/28 fractions for T2N0, 54 Gy/30 fractions for T3/4Nany	5-FU and MMC	28.6	UV Cox, MV Cox	Fair
15	Bitterman et al. (2015) [34]	SC, NA	109	2004–2013	IMRT (60%) 3D-CRT (40%)	45 Gy + in 1.8 Gy/fraction	5-FU and MMC	14.9	Log-rank, MV Cox	Good
16	Fraunholz et al. (2013) [35]	MC, EU	103	1989–2011	3D-CRT	50.4 Gy in 1.8 or 2 Gy/fraction	5-FU and MMC	44.0	Log-rank, MV Cox	Good
17	Schernberg et al. (2017)^a^ [36]	SC, EU	103	2006–2016	IMRT (53%) 3D-CRT (47%)	45 Gy/25 fractions of 1.8 Gy or 44 Gy/22 fractions of 2 Gy	5-FU and MMC or Cap	38.7	Log-rank, MV Cox	Good
18	Hosni et al. (2018) [37]	SC, NA	101	2008–2013	IMRT	45 Gy/25 fractions for T1N0, 54 Gy/30 fractions for T1/2 N + or 63 Gy/35 fractions for T3/4Nany	5-FU and MMC	56.5	UV Cox, MV Cox	Fair
19	Oblak et al. (2016) [38]	SC, EU	100	2003–2013	3D-CRT IMRT	45 Gy/25 fractions	5-FU and MMC or Cap	52.0	Log-rank, MV Cox	Good

^**a**^used to differentiate between two studies by the same author published in the same year. *MC* multi-centre, *SC* single-centre, *EU* Europe, *NA* North America, *IN* International, *NR* not reported, *Gy* Gray, *MMC* mitomycin C, *Cap* capecitabine, *5-FU* 5-fluorouracil, *Cisp* cisplatin

*UV* univariable, *BV* bivariable, *MV* multivariable, *Cox* Cox regression, *Log-rank* log-rank statistical test

### Outcomes

Outcome definitions varied considerably. Appendix D presents the definitions extracted from each study and how they were categorised. Nine outcome categories were used: three disease activity (freedom-from-disease, locoregional failure (LRF) and distant failure) as well as six survival categories (overall survival (OS), disease-free survival (DFS), colostomy-free survival (CFS), cancer-specific survival, local failure-free survival and metastasis-free survival (MFS)). Disease-free survival and progression-free survival were grouped together, as definitions overlapped in most papers. Local and regional failures were grouped with locoregional failures, due to the small number of studies reporting only on the latter. Freedom-from-disease, a category which was not included in CORMAC, was devised in order to include definitions of time-to-recurrence, time-to-failure (not specified as local, regional or distant) and disease-free survival where death was not considered an event. Commonly investigated outcomes were OS (*n* = 17), LRF (*n* = 11) and DFS (*n* = 11). Appendix E lists all outcomes reported, along with all factors tested.

### Clinical prognostic factors

Table 2 presents clinical factors identified as prognostic for each outcome in more than one study, categorised by UVA and MVA. For prognostic factors identified in MVA, the range of factor effects (HRs) across studies is also reported. Eight unique prognostic factors were established by more than one study in UVA and seven in MVA (See Appendix F for full results).

**Table 2:** Clinical factors identified as prognostic for worse outcomes by more than one study

**Univariable analysis**				
**Outcome** **(number of studies reporting outcome)**	**Factor**	**Times identified as prognostic**	**Total times tested**	**Studies which identified factor as prognostic**
**Overall survival** **(*n* = 17)**	Higher N stage	10	16	[15, 21, 22, 25–28, 35, 36, 38]
	Higher T stage	9	16	[15, 21, 22, 27, 28, 35–38]
	Male sex	7	12	[15, 21, 25, 27–29, 37]
	Worse performance status	3	4	[15, 29, 38]
	Older age	3	4	[24, 27, 37]
	Incomplete/interrupted RT or breaks	2	2	[15, 24]
	Longer CRT duration	2	5	[36, 38]
**Locoregional failure** **(*n* = 11)**	Higher N stage	7	11	[15, 21, 26–28, 30, 38]
	Higher T stage	7	11	[15, 21, 26–28, 32, 38]
	Male sex	5	9	[15, 21, 27–29]
	Worse performance status	4	4	[15, 24, 29, 38]
	Longer CRT duration	2	2	[32, 38]
**Disease-free survival (*n* = 11)**	Male sex	5	8	[21, 27, 29, 30, 37]
	Higher N stage	4	9	[21, 22, 27, 30]
	Higher T stage	4	10	[21, 22, 28, 37]
**Metastasis-free survival** **(*n* = 5)**	Higher T stage	5	5	[21, 22, 30, 35, 36]
	Higher N stage	4	4	[21, 30, 35, 36]
	Male sex	2	4	[21, 30]
**Freedom from disease** **(*n* = 4)**	Higher N stage	4	4	[15, 28, 31, 38]
	Male sex	2	3	[15, 28]
	Higher T stage	2	3	[15, 38]
**Colostomy-free survival** **(*n* = 4)**	Higher T stage	3	4	[22, 26, 37]
**Cancer-specific survival** **(*n* = 3)**	Higher T stage	2	3	[35, 38]
	Higher N stage	2	3	[35, 38]
**Multivariable analysis**				
**Outcome** **(number of studies reporting outcome)**	**Factor**	**Times identified as prognostic**	**Factor effect range (HR)**	**Studies which identified factor as prognostic**
**Overall survival** **(*n* = 17)**	Male sex	7	1.92 – 4.80	[15, 21, 25, 27–29, 37]
	Higher T stage	3	2–88 – 4.98	[22, 34, 37]
	Older age	3	1.05 – 2.43	[24, 37]
	Higher N stage	3	1.88 – 5.80	[25, 26, 36]
	Higher AJCC stage	2	2.23 – 2.82	[22, 38]
**Locoregional failure** **(*n* = 11)**	Male sex	4	2.08 – 3.40	[15, 21, 27, 29]
	Higher N stage	3	2.23 – 3.58	[15, 21, 30]
	Incomplete/interrupted RT or breaks	2	2.47 – 4.96	[15, 22]
	Worse performance status	2	3.82 – 5.50	[24, 29]
**Disease-free survival** **(*n* = 11)**	Male sex	4	2.13 – 3.60	[21, 27, 29, 37]
	Higher T stage	3	2.57 – 7.02	[22, 23, 37]
	Higher N stage	2	N/A*	[21, 23]
**Metastasis-free survival** **(*n* = 5)**	Male sex	2	3.87 – 4.08	[21, 23]
	Higher T stage	2	2.61 – 3.54	[21, 22]
	Higher N stage	2	2.41 – 4.49	[21, 30]
**Freedom from disease** **(*n* = 4)**	Male sex	2	2.16 – 2.16	[15, 28]
**Colostomy-free survival** **(*n* = 4)**	Higher T stage	3	3.65 – 4.10	[22, 26, 37]

These clinical factors were identified through univariable and multivariable analysis, and were stratified by outcome. A number of studies reported on “gender”, however this was analysed in conjunction with “sex” throughout the study, since “sex” is used when reporting on biological factors instead of gender identity, or psychosocial or cultural factors. *HR* Hazard Ratio, *N/A* Not available. *Factor effects (HRs) were provided by only one study for this prognostic factor, therefore the effect range could not be determined

In UVA, T stage, N stage and sex were the most commonly tested factors for all seven outcomes for which prognostic factors were identified (Table 1). T stage was prognostic for all outcomes; in 56% of the studies that tested it for OS, in 64% for LRF, in 40% for DFS, in 100% for MFS, in 67% for freedom-from-disease, in 75% for CFS and in 67% for cancer-specific survival. Similarly, N stage was prognostic for six of seven outcomes. It was prognostic in 63% of the studies testing for OS, in 64% for LRF, in 44% for DFS, in 100% for MFS, in 100% for freedom-from-disease and in 67% for cancer-specific survival. The third most identified prognostic factor in UVA was sex. It was prognostic for five of the seven outcomes, in 58% of the studies that tested it for OS, in 56% for LRF, in 63% for DFS, in 50% for MFS and in 67% for freedom-from-disease. Performance status was also identified as prognostic in 75% of the studies that tested it for OS, and in 100% of studies that tested it for LRF.

In MVA, sex retained its prognostic significance, appearing as the predominant prognostic factor for six of the seven outcomes, altogether identified in nine studies [15, 21, 22, 25, 27–29, 35, 37]. Other commonly identified prognostic factors included higher T stage (OS, DFS, MFS and CFS; identified in seven studies [21–23, 26, 28, 34, 37]) and higher N stage (OS, LRF, DFS, MFS; identified in seven studies [15, 21, 23, 25, 26, 30, 36]). The rest of the factors were identified as prognostic for a single outcome only; age and AJCC stage for OS, as well as incomplete/interrupted radiotherapy and performance status for LRF.

### Biomarkers and imaging prognostic factors

A smaller number of studies (*n* = 8) examined the prognostic significance of biomarkers [25, 27–30, 35, 36, 38]. Only four unique biomarkers were deemed prognostic overall by more than one study in both UVA and MVA (Table 3 and Appendix G).

**Table 3:** Biomarkers identified as prognostic for worse outcomes by more than one study

**Univariable analysis**				
**Outcome** **(number of studies reporting outcome)**	**Factor**	**Times identified as prognostic**	**Total times tested**	**Studies which identified factor as prognostic**
**Overall survival** **(*n* = 17)**	Lower HPV16 load	2	3	[27, 28]
	Neutrophilia	2	2	[29, 36]
	Anaemia	2	2	[29, 36]
**Locoregional failure** **(*n* = 11)**	Lower HPV16 load	2	3	[27, 28]
**Disease-free survival** **(*n* = 11)**	Leukocytosis	2	2	[29, 36]
	Neutrophilia	2	2	[29, 36]
**Multivariable analysis**				
**Outcome** **(number of studies reporting outcome)**	**Factor**	**Times identified as prognostic**	**Factor effect range (HR)**	**Studies which identified factor as prognostic**
**Overall survival** **(*n* = 17)**	Leukocytosis	2	4.60 – 19.90	[29, 36]
	Neutrophilia	2	4.40 – 22.70	[29, 36]
**Locoregional failure** **(*n* = 11)**	Lower HPV16 load	2	3.57 – 4.51	[27, 28]
**Disease-free survival** **(*n* = 11)**	Leukocytosis	2	6.90 – 7.10	[29, 36]
	Neutrophilia	2	5.00 – 7.60	[29, 36]
	Anaemia	2	2.50 – 5.30	[29, 36]

These biomarkers were identified through univariable and multivariable analysis and were stratified by outcome. *HPV* human papillomavirus, *HR* Hazard ratio

In UVA, HPV16 load from pre-treatment biopsies was found to be prognostic for OS (2/3 – 67% of studies [27, 28]) and for LRF (2/3 – 67% of studies [27, 28]), whereas the presence of baseline neutrophilia (circulating blood neutrophil count of more than 7500/mm3 in one study and more than 7G/L in the second study) was found to be prognostic for OS (2/2 – 100% of studies [29, 36]) and DFS (2/2 – 100% of studies [29, 36]). Additionally, baseline anaemia (haemoglobin count < 13 g/dL) was deemed prognostic for OS only (2/2 – 100% of studies [29, 36]) and the presence of baseline leukocytosis markers (white blood cell count > 10,000/mm3 in one study and more than 10G/L in the second study) for DFS only.

In MVA, baseline neutrophilia retained its prognostic significance for both OS (two studies [29, 36]) and DFS (two studies [29, 36]), whereas HPV16 load retained its prognostic significance for LRF (two studies [27, 28]) only. Baseline leukocytosis was found to be prognostic for DFS (two studies [29, 36]) and for OS (two studies [29, 36]). Lastly, baseline anaemia was identified as prognostic for DFS (two studies [29, 36]) only.

Only two studies [23, 33] investigated imaging-related prognostic factors. In UVA, one study [33] identified post-treatment PET-CT SUVmax (positron emission tomography and computed tomography maximum standardized uptake value) and change in SUVmax (pre- vs. post-treatment) to be prognostic for OS. The pre-treatment and post-treatment SUVmax values were both found to be prognostic for local failure-free survival. In MVA, the post-treatment SUVmax and the change in SUVmax retained prognostic significance for OS. In the second study [23], a selection of radiomics markers were identified as prognostic for DFS (Appendix H). For local failure-free survival, only the high post-treatment SUVmax was deemed prognostic in MVA (Appendix H).

## Discussion

This systematic review summarises the findings from studies examining prognostic factors for anal cancer outcomes following CRT with contemporary conformal radiotherapy techniques. By limiting our findings to studies with cohorts treated with conformal radiotherapy techniques, we aimed to ensure that the prognostic factors identified are the most informative to current practice and are representative of the more prevalent HPV-driven biology and the higher survival rates which have been observed in the past few years. N stage, T stage, and sex were established as the most prevalent and reliable clinical prognostic factors for the majority of outcomes explored, in both UVA and MVA. Few biomarkers have been identified as prognostic by more than one study: pre-treatment biopsy HPV load, as well as the presence of leukocytosis, neutrophilia and anaemia at baseline measurement. The review also highlighted the lack of studies with large cohorts exploring the prognostic significance of imaging factors.

Due to the rarity of anal cancer, only few randomised prospective clinical trials have been conducted to date; none of which have employed conformal radiotherapy techniques and reported on prognostic factors. Reports from randomised trials using non-conformal radiotherapy techniques support the prognostic role of N stage, T stage and sex [3, 39]. Male sex and a higher N stage were found to be strong prognostic indicators for worse OS [3, 40, 41], for higher risk of local failure [3, 42] and LRF [41]. The prognostic role of T stage was less apparent, since higher T stage was only found to be prognostic for worse OS [40] and local failure [42]. Our results suggest that a higher T stage is prognostic for higher risk of LRF in UVA, but not in MVA. Although the aforementioned trials used highly standardised approaches and studied a relatively large number of patients, crude radiotherapy techniques were employed, therefore the prescribed and received radiotherapy doses are likely to differ significantly [43].

In terms of tumour biomarkers, HPV status is the strongest previously-established prognostic indicator in anal cancer [44, 45]. A previous study [46] also established the prognostic significance of p16^INK4A^ in anal cancer, a biomarker commonly used as a surrogate for HPV involvement. In line with these findings, our results confirm the prognostic role of pre-treatment biopsy HPV load in anal cancer. Treatment modification based on HPV status is currently being tested in a head and neck cancer clinical trial, where treatment is stratified based on the HPV status of the cancer [47]. Apart from HPV load, no other tumour biomarkers were identified as prognostic in this review. In terms of haematological biomarkers, long-term outcome data from the ACT1 randomised controlled trial reported that a higher baseline white blood cell count is prognostic for worse OS [41], supporting our results (Table 3). Baseline anaemia, another haematological biomarker identified as prognostic in our review, may carry important clinical implications. Although not predictive of OS in the ACT1 data, it was independently predictive of anal cancer death. In cervical cancer, another HPV-driven cancer, blood transfusions are given if haemoglobin levels are below 10 g/dl prior to CRT and this may be an area of future clinical consideration in anal cancer treatment.

Due to the lack of studies exploring imaging factors, it is difficult to put our review findings into perspective. Future radiomics research in this setting should focus on multicentre cohorts; but we also noted the lack of secondary or explorative radiomics research from prospective trials. Further research in this area may for instance help identify tumour volumes of greater radiotherapy resistance for boosting.

Three other reviews have previously investigated prognostic factors for anal cancer. One systematic review focused solely on biomarkers and did not include any information on general, pathological or treatment-related prognostic factors [48]. A second systematic review examined the prognostic factors for the specific subset of HIV-positive anal cancer patients undergoing highly active antiretroviral therapy (HAART) [49]. The third review [50] explored clinical, treatment-related as well as molecular prognostic factors, but was a narrative rather than a systematic review. None focused specifically on identifying prognostic factors for outcomes after conformal radiotherapy.

The current work has several limitations. As anal cancer is rare, reports exploring this topic are often single-centre studies with small cohorts, meaning that the power to identify relevant prognostic factors, especially factors with relatively limited effect size or with low prevalence, may be limited. Any factors identified and their effect estimates may suffer from small sample bias [51]. We opted for a sample size of 100 patients as the cut-off point, following an initial screen of available studies, in order to ensure that a reasonable number of studies could be included in the final analysis and the factors identified were generalisable. Through the initial screen, only 43 studies which had cohorts of more than 20 patients were identified. If studies with 20–100 patients had been included, seven additional studies exploring biomarkers and 12 additional studies exploring imaging factors would have been considered, and a larger number of factors would potentially be identified as prognostic. Only few of the studies included in this review distinguished between cancers of the anal canal and perianal cancers (Appendix C). Therefore, it was not possible to identify prognostic factors for a specific tumour location or subtype. Additionally, the TNM staging version used varied from the 6^th^ edition to the 8^th^ edition across studies (Appendix C) and some studies did not report the version used at all. As a result, in this review all tumour and nodal staging information was analysed together, without accounting for the version used.

There was large variation in treatment regimens, factors tested and outcome definitions between studies. This renders the identification of prognostic factors for anal cancer challenging and highlights the need for uniform outcome definitions, not only in clinical trials and research, but also in routine clinical practice [52]. The studies themselves suffer from several limitations as well, especially in the statistical methodology. The majority of studies applied a univariable screening technique to select factors for MVA. Generally, univariable screening should be avoided for such analyses, as it invalidates the effect and significance estimates in MVA [53, 54], and more robust approaches should be used instead [53, 55]. Moreover, a considerable number of studies did not report on factor effects acquired from UVA or MVA, therefore we could not summarise factor effects across studies. Since a meta-analysis could not be conducted, only a summary of factor effects is reported in this review. Lastly, the proportion of times each factor was identified as prognostic, which is a better indicator of the reliability of the prognostic significance of a factor, could not be calculated from MVA results, due to a lack of detail about the total number of times each factor was tested for each outcome.

Overall, this study confirms the prognostic value of only few well-established clinical factors and biomarkers relevant to contemporary clinical practice. No novel prognostic factors have been identified. This emphasises the lack of studies with large cohorts treated with conformal radiotherapy that report on prognostic factors, especially studies exploring biomarkers and imaging factors. In spite of the remarkable advances in anal cancer treatment efficacy and the reduction of toxicity through conformal CRT, our understanding of the biomarker and imaging factors that predict the outcomes of this disease is still very limited. To tackle the challenge of prognostic factor identification, larger multi-institutional studies and prospective clinical trials would need to be conducted, not only on a national scale, but also on an international scale using approaches that link data across borders [56].

## Conclusions

This systematic review confirms the following prognostic factors for outcomes following anal cancer treatment with conformal CRT: T stage, N stage, sex, pre-treatment biopsy HPV load, as well as the presence of baseline leukocytosis, neutrophilia and anaemia. The prognostic information presented can be used as a starting point for variable selection in future prognostic modelling studies. Additionally, by establishing a set of prognostic and potentially predictive factors for anal cancer outcomes, we may be able to stratify patients into risk groups in order to design more personalised clinical trials in the future. Radiotherapy dose modification based on risk by T and N stage is being evaluated in the currently recruiting PLATO clinical trial [17], with translational research into prognostic biomarkers and imaging embedded within the trial design. This will in turn provide us with greater insight into how to effectively treat this disease using a more personalised approach.

## Supplementary Information

**Additional file 1: Appendix A. **Full search strategies employed in Embase and Medline to identify relevant papers between January 1st 2000 and June 30th 2020. **Appendix B.** Complete results from the study quality appraisal by both reviewers (ST and RS), including the assessment criteria used. Y: Yes. N: No. NR: Not reported. **Appendix C.** Complete overview of study characteristics, including the predictors tested in each study. NR: not reported. SCC: Squamous cell carcinoma. RT: radiotherapy. CRT: chemoradiotherapy. MMC: Mitomycin C. **Appendix D.** Outcome definitions given in each study, stratified into nine categories. The final stratification yielded three disease activity outcome categories and six survival outcome categories. **Appendix E.** All outcomes reported in each study, along with all factors tested in both univariable and multivariable analysis. **Appendix F.** Clinical factors identified as prognostic for worse outcomes through univariable and multivariable analysis, stratified by outcome. Where available, factor effects and parameterisation used for analysis are also included. **Appendix G.** Biomarkers identified as prognostic for worse outcomes through univariable and multivariable analysis, stratified by outcome. Where available, factor effects and parameterisation used for analysis are also included. **Appendix H.** Imaging factors identified as prognostic for worse outcomes through univariable and multivariable analysis, stratified by outcome. Where available, factor effects are also included.

## Data Availability

All data generated or analysed during this study are included in this published article [and its supplementary information files].

## Abbreviations

5-FU: 5-Fluorouracil
ASCC: Anal squamous cell carcinoma
CRT: Chemoradiotherapy
CFS: Colostomy-free survival
CT: Computed tomography
DFS: Disease-free survival
HR: Hazard ratio
HPV: Human papillomavirus
IMRT: Intensity modulated radiotherapy
LRF: Locoregional failure
SUVmax: Maximum standardized uptake value
MFS: Metastasis-free survival
MMC: Mitomycin C
MVA: Multivariable analysis
NIH: National Institutes of Health
OAR: Organs at risk
OS: Overall survival
PET: Positron emission tomography
UVA: Univariable analysis
VMAT: Volumetric arc therapy
