# Supplementary material for: Prognostic factors for patients with anal cancer treated with conformal radiotherapy—a systematic review
Source: BMC Cancer. 2022 Jun 3;22:607. doi: 10.1186/s12885-022-09729-4 (PMC9164501; doi:10.1186/s12885-022-09729-4)
Supplement: Supplementary file 1 — Additional file 1: Appendix A. Full search strategies employed in Embase and Medline to identify relevant papers between January 1st 2000 and June 30th 2020. Appendix B. Complete results from the study quality appraisal by both reviewers (ST and RS), including the assessment criteria used. Y: Yes. N: No. NR: Not reported. Appendix C. Complete overview of study characteristics, including the predictors tested in each study. NR: not reported. SCC: Squamous cell carcinoma. RT: radiotherapy. CRT: chemoradiotherapy. MMC: Mitomycin C. Appendix D. Outcome definitions given in each study, stratified into nine categories. The final stratification yielded three disease activity outcome categories and six survival outcome categories. Appendix E. All outcomes reported in each study, along with all factors tested in both univariable and multivariable analysis. Appendix F. Clinical factors identified as prognostic for worse outcomes through univariable and multivariable analysis, stratified by outcome. Where available, factor effects and parameterisation used for analysis are also included. Appendix G. Biomarkers identified as prognostic for worse outcomes through univariable and multivariable analysis, stratified by outcome. Where available, factor effects and parameterisation used for analysis are also included. Appendix H. Imaging factors identified as prognostic for worse outcomes through univariable and multivariable analysis, stratified by outcome. Where available, factor effects are also included. [file 12885_2022_9729_MOESM1_ESM.pdf]

Appendix A. Full search strategies employed in Embase and Medline to identify relevant papers between January 1st 2000 and June 30th 2020.

Table A1. Complete search strategy used in Embase.

**Database: Embase Classic+Embase <1947 to 2020 June 30>**	
1	exp radiotherapy/ (599698)
2	Radiation Oncology/ (3434)
3	(radiotherap* or radiotreat* or roentgentherap* or radiosurg*).tw. (285655)
4	((radiat* or radio* or irradiat* or roentgen or x-ray or xray) adj4 (therap* or treat* or repair* or oncolog* or surg*)).tw. (396128)
5	(RT or RTx or XRT).tw. (308595)
6	exp chemoradiotherapy/ (52074)
7	(chemoradiotherap* or radiochemotherap* or chemoradiation*).tw. (53407)
8	(CRT or CRTx or CCRT or NCRT or RCTx or RT-CT or chemoRT).tw. (38794)
9	or/1-8 [radiotherapy or chemeradiotherapy] (1107122)
10	exp Anus cancer/ (8197)
11	((anus or anal) adj5 (cancer* or neoplas* or carcinoma* or tumo?r*)).tw,kw. (10640)
12	or/10-11 [anal cancer] (13164)
13	(predict* and (outcome* or risk* or model*)).tw. (1210603)
14	(validate or rule*).tw. (376396)
15	predict*.ti. (470931)
16	((history or variable* or criteria or scor* or characteristic* or finding* or factor*) and (predict* or model* or decision* or identify or prognose)).tw. (3153437)
17	(prognostic and (history or variable* or criteria or scor* or characteristic* or finding* or factor* or model*)).tw. (343683)
18	ROC Curve/ (57455)
19	(stratification or discrimination or discriminate or c-statistic or c statistic or area under the curve or AUC or calibration or indices or algorithm or multivariable or (model and outcome) or classif*).tw. (2013800)
20	((model* or clinical).tw. or logistics models/) and decision.tw. (183621)
21	or/13-20 [predictive factor or outcomes] (5491959)
22	9 and 12 and 21 [radiotherapy or chemoradiotherapy and anal cancer and predictive factors for outcomes] (1219)
23	limit 22 to yr="2000 -Current" (1134)
24	remove duplicates from 23 (1109)

Table A2. Complete search strategy used in Medline.

**Database: Ovid MEDLINE(R) and Epub Ahead of Print, In-Process & Other Non-Indexed Citations and Daily <1946 to June 30, 2020>**	
1	exp Radiotherapy/ (184934)
2	Radiation Oncology/ (4114)
3	(radiotherap* or radiotreat* or roentgentherap* or radiosurg*).tw. (179332)
4	((radiat* or radio* or irradiat* or roentgen or x-ray or xray) adj4 (therap* or treat* or repair* or oncolog* or surg*)).tw. (244503)
5	(RT or RTx or XRT).tw. (201228)
6	exp Chemoradiotherapy/ (14534)

7	(chemoradiotherap* or radiochemotherap* or chemoradiation*).tw. (30229)
8	(CRT or CRTx or CCRT or NCRT or RCTx or RT-CT or chemoRT).tw. (18166)
9	or/1-8 [radiotherapy or chemeradiotherapy] (615114)
10	exp Anus Neoplasms/ (6335)
11	((anus or anal) adj5 (cancer* or neoplas* or carcinoma* or tumo?r*)).tw,kw. (6355)
12	or/10-11 [anal cancer] (9210)
13	(predict* and (outcome* or risk* or model*)).tw. (847317)
14	(validate or rule*).tw. (265142)
15	predict*.ti. (325883)
16	((history or variable* or criteria or scor* or characteristic* or finding* or factor*) and (predict* or model* or decision* or identify or prognose)).tw. (2253192)
17	(prognostic and (history or variable* or criteria or scor* or characteristic* or finding* or factor* or model*)).tw. (216359)
18	ROC Curve/ (57771)
19	(stratification or discrimination or discriminate or c-statistic or c statistic or area under the curve or AUC or calibration or indices or algorithm or multivariable or (model and outcome) or classif*).tw. (1421211)
20	((model* or clinical).tw. or logistics models/) and decision.tw. (123422)
21	or/13-20 [predictive factor or outcomes] (3986478)
22	9 and 12 and 21 [radiotherapy or chemoradiotherapy and anal cancer and predictive factors for outcomes] (522)
23	limit 22 to yr="2000-2020" (458)

Appendix B. Complete results from the study quality appraisal by both reviewers (ST and RS), including the assessment criteria used. Y: Yes. N: No. NR: Not reported.

Case series study appraisal criteria:
1. Was the study question or objective clearly stated?
2. Was the study population clearly and fully described, including a case definition?
3. Were the cases consecutive?
4. Were the subjects comparable? Reasonably homogeneous study population
5. Was the intervention clearly described?
6. Were the outcome measures clearly defined, valid, reliable, and implemented consistently across all study participants?
7. Was the length of follow-up adequate? 3 years according to PLATO
8. Were the statistical methods well-described?
9. Were the results well-described?

Table B1. Study quality appraisal by ST.

Study/Criterion	1	2	3	4	5	6	7	8	9	Quality rating (Good/Fair/Poor)	Type of study
Shakir et al. (2020)	Y	Y	Y	Y	Y	Y	N	Y	Y	Good	Case series
Martin et al. (2020)	Y	Y	NR	Y	Y	Y	Y	Y	Y	Good	Case series
de Bellefon et al. (2020)	N	Y	Y	Y	Y	Y	Y	Y	Y	Good	Case series
Brown et al. (2019)	Y	Y	Y	Y	N	Y	N	Y	Y	Good	Case series
Rouard et al. (2019)	Y	Y	Y	Y	Y	Y	N	Y	Y	Good	Case series
Franco et al. (2018)	Y	Y	NR	Y	Y	Y	N	Y	Y	Good	Case series
Call et al. (2016)	N	Y	NR	Y	Y	Y	N	Y	Y	Fair	Case series
Balermpas et al. (2017)	N	Y	NR	Y	Y	Y	Y	Y	Y	Good	Case series
Rodel et al. (2018)	N	Y	NR	Y	Y	Y	Y	Y	Y	Good	Case series
Schernberg et al. (2017)	Y	Y	Y	Y	Y	Y	Y	Y	Y	Good	Case series
Martin et al. (2019)	Y	Y	NR	Y	Y	Y	NR	Y	Y	Good	Case series
Oehler-Janne et al. (2008)	Y	Y	Y	N	Y	Y	Y	Y	Y	Good	Case series
Susko et al. (2020)	Y	Y	NR	Y	Y	Y	N	Y	Y	Good	Case series
Cardenas et al. (2017)	Y	Y	NR	Y	Y	N	N	Y	Y	Fair	Case series
Bitterman et al. (2015)	N	Y	Y	Y	Y	Y	N	Y	Y	Good	Case series
Fraunholz et al. (2013)	Y	Y	NR	Y	Y	Y	Y	Y	Y	Good	Case series
Schernberg et al. (2017)*	Y	Y	Y	Y	Y	N	Y	Y	Y	Good	Case series
Hosni et al. (2018)	N	Y	NR	Y	Y	N	Y	Y	Y	Fair	Case series
Oblak et al. (2016)	Y	Y	Y	Y	Y	Y	Y	Y	Y	Good	Case series

Table B2. Study quality appraisal by RS.

Study/Criterion	1	2	3	4	5	6	7	8	9	Quality rating (Good/Fair/Poor)	Type of study
Shakir et al. (2020)	Y	Y	Y	Y	Y	Y	N	Y	Y	Good	Case series
Martin et al. (2020)	Y	Y	NR	Y	Y	Y	Y	Y	Y	Good	Case series
de Bellefon et al. (2020)	Y	Y	Y	Y	Y	Y	Y	Y	Y	Good	Case series
Brown et al. (2019)	Y	Y	Y	Y	Y	Y	N	Y	Y	Good	Case series
Rouard et al. (2019)	Y	Y	Y	Y	Y	Y	N	Y	Y	Good	Case series
Franco et al. (2018)	Y	Y	NR	Y	Y	Y	N	Y	Y	Good	Case series

Call et al. (2016)	Y	Y	NR	Y	Y	Y	N	Y	Y	Good	Case series
Balermpas et al. (2017)	N	Y	NR	Y	Y	Y	Y	Y	Y	Good	Case series
Rodel et al. (2018)	N	Y	NR	Y	Y	Y	Y	Y	Y	Good	Case series
Schernberg et al. (2017)	Y	Y	Y	Y	Y	Y	Y	Y	Y	Good	Case series
Martin et al. (2019)	Y	Y	NR	Y	Y	Y	NR	Y	Y	Good	Case series
Oehler-Janne et al. (2008)	N	Y	Y	N	Y	Y	Y	Y	Y	Good	Case series
Susko et al. (2020)	Y	Y	Y	Y	Y	N	N	Y	Y	Good	Case series
Cardenas et al. (2017)	Y	Y	NR	Y	Y	N	N	Y	Y	Fair	Case series
Bitterman et al. (2015)	Y	Y	Y	Y	Y	Y	N	Y	Y	Good	Case series
Fraunholz et al. (2013)	Y	Y	NR	Y	Y	Y	Y	Y	Y	Good	Case series
Schernberg et al. (2017)*	Y	Y	Y	Y	Y	N	Y	Y	Y	Good	Case series
Hosni et al. (2018)	N	Y	NR	Y	Y	N	Y	Y	Y	Fair	Case series
Oblak et al. (2016)	Y	Y	Y	Y	Y	Y	Y	Y	Y	Good	Case series

Appendix C. Complete overview of study characteristics, including the predictors tested in each study. NR: not reported. SCC: Squamous cell carcinoma. RT: radiotherapy. CRT: chemoradiotherapy. MMC: Mitomycin C.

#	Study	Year of publication	Location	Study design	Number of patients	Years of treatment	RT technique	Cancer subtype and location in cohort	TNM staging version used	Median follow-up (months)	Type of statistical analysis used	Predictors tested	Quality score
**1**	Patterns and Predictors of Relapse Following Radical Chemoradiation Therapy Delivered Using Intensity Modulated Radiation Therapy with a Simultaneous Integrated Boost in Anal Squamous Cell Carcinoma [Shakir et al. (2020)]	2020	Multi-centre, Europe	Retrospective	385	2013-2018	IMRT	SCC of the anal canal and anal margin	7 & 8	24.0	Univariable Cox regression, multivariable Cox regression	Age, sex, performance status, T stage, N stage (TNM 7 and 8), RT completion, chemotherapy type	Good
**2**	Acute organ toxicity correlates with better clinical outcome after chemoradiotherapy in patients with anal carcinoma [Martin et al. (2020)]	2020	Single centre, Europe	Retrospective	223	1996-2017	3D-CRT (58%) and IMRT (42%)	SCC (location not specified)	7	46.0	Univariable Cox regression, multivariable Cox regression	T stage, N stage, age, sex, high grade acute organ toxicity (HGAOT)	Good
**3**	Long-term follow-up experience in anal canal cancer treated with Intensity-Modulated Radiation Therapy: Clinical outcomes, patterns of relapse and predictors of failure [de Bellefon et al. (2020)]	2020	Single centre, Europe	Retrospective	193	2005-2017	IMRT	SCC of the anal canal	7 & 8	70.0	Univariable Cox regression, multivariable Cox regression	Only significant factors reported - T stage, N stage, AJCC stage, sex, RT breaks, exclusive RT, lack of MMC, residual disease	Good
**4**	Prediction of outcome in anal squamous cell carcinoma using radiomic feature analysis of pre-treatment FDG PET-CT [Brown et al. (2019)]	2019	Single centre, Europe	Retrospective	189	2008-2016	2D/ 3D-CRT (79%) and VMAT (21%)	SCC (location not specified)	NR	35.1	Multivariable logistic regression	Only significant factors reported - Multiple FDG-PET scan variables, sex, age, T stage, N stage	Good

**5**	Intensity-modulated radiation therapy of anal squamous cell carcinoma: Relationship between delineation quality and regional recurrence [Rouard et al. (2019)]	2019	Multi-centre, Europe	Retrospective	165	2006-2016	IMRT	SCC of the anal canal and anal margin	NR	33.8	Bivariable Cox regression, multivariable Cox regression	Sex, age, performance status, immunodepression, smoking status, tumour localisation, T stage, N stage, location of involved lymph nodes, tumour size, keratinisation, differentiation, HPV status, RT breaks, tumour boost technique, tumour total dose, chemotherapy type, multiple delineation variables	Good
**6**	The prognostic role of hemoglobin levels in patients undergoing concurrent chemo-radiation for anal cancer [Franco et al. (2018)]	2018	Multi-centre, Europe	Retrospective	161	NR	IMRT	SCC of the anal canal and anal margin	NR	27.0	Log-rank analysis, univariable Cox regression, multivariable Cox regression	Age, sex, T stage, N stage, response to treatment, overall treatment duration, RT total dose, boost, OTT, basal haemoglobin levels	Good
**7**	Intensity-modulated Radiation Therapy for Anal Cancer Results From a Multi-Institutional Retrospective Cohort Study [Call et al. (2016)]	2016	Multi-centre, North America	Retrospective	152	NR	IMRT	SCC (location not specified)	NR	26.8	Log-rank analysis, multivariable Cox regression	Dose, N stage, T stage, RT duration	Fair
**8**	Human papilloma virus load and PD-1/PD-L1, CD8+ and FOXP3 in anal cancer patients treated with chemoradiotherapy: Rationale for immunotherapy [Balermpas et al. (2017)]	2017	Multi-centre, Europe	Retrospective	150	NR	3D-CRT and IMRT	SCC (location not specified)	NR	40.0	Log-rank analysis, multivariable Cox regression	Age, gender, T stage, N stage, grade, HPV load, and CD8, PD1, PD-L1, FOXP3, pCASP8 expression	Good
**9**	Prognostic impact of RITA expression in patients with anal squamous cell carcinoma treated with chemoradiotherapy [Rodel et al. (2018)]	2018	Multi-centre, Europe	Retrospective	140	NR	3D-CRT and IMRT	SCC (location not specified)	NR	40.0	Log-rank analysis, multivariable Cox regression	Gender, T stage, N stage, HPV-16 DNA load, RITA expression	Good

**10**	External validation of leukocytosis and neutrophilia as a prognostic marker in anal carcinoma treated with definitive chemoradiation [Schernberg et al. (2017)]	2017	Multi-centre, Europe	Retrospective	133	2000-2015	IMRT (77%) and 3D-CRT (23%)	SCC (location not specified)	7	37.4	Log-rank analysis, multivariable Cox regression	Age, sex, T stage, N stage, performance status, leukocytosis, neutrophilia, anaemia, lymphopenia, monocytosis, thrombocytosis	Good
**11**	C-Reactive Protein-to-Albumin Ratio as Prognostic Marker for Anal Squamous Cell Carcinoma Treated with Chemoradiotherapy [Martin et al. (2019)]	2019	Single centre, Europe	Retrospective	126	2004-2016	IMRT (65%) and 3D-CRT (35%)	SCC (location not specified)	7	NA	Log-rank analysis, univariable Cox regression, multivariable Cox regression	Age, sex, HIV status, T stage, N stage, grade, C reactive protein to Albumin ratio, RT modality, RT total dose	Good
**12**	HIV-specific differences in outcome of squamous cell carcinoma of the anal canal: a multicentric cohort study of HIV-positive patients receiving highly active antiretroviral therapy [Oehler-Janne et al. (2008)]	2008	Multi-centre, International	Retrospective	121	1997-2006	3D-CRT	SCC of the anal canal	NR	36.0	Log-rank analysis, univariable Cox regression, multivariable Cox regression	Not explicitly reported - Age, sex, WHO performance status, histologic subtype, tumour size, N stage, M stage, CDC stage, CD4 count, viral load, HAART type	Good
**13**	Factors Impacting Differential Outcomes in the Definitive Radiation Treatment of Anal Cancer Between HIV-Positive and HIV-Negative Patients [Susko et al. (2020)]	2020	Single centre, North America	Retrospective	111	2005-2018	3D-CRT and IMRT	SCC (location not specified)	NR	28.0	Log-rank analysis, univariable Cox regression, multivariable Cox regression	Age, sex, T stage, N stage, HIV status, time from diagnosis to treatment, treatment duration	Good
**14**	Quantitative FDG-PET/CT predicts local recurrence and survival for squamous cell carcinoma of the anus [Cardenas et al. (2017)]	2017	Single centre, North America	Retrospective	110	2003-2013	IMRT (75%) and 2D-CRT (25%)	SCC (location not specified)	NR	28.6	Univariable Cox regression, multivariable Cox regression	Multiple FDG-PET scan variables, RT modality, chemotherapy, T stage, N stage, HIV status	Fair
**15**	Comparison of anal cancer outcomes in public and private hospital patients treated at a single radiation oncology center [Bitterman et al. (2015)]	2015	Single centre, North America	Retrospective	109	2004-2013	IMRT (60%) and 3D-CRT (40%)	SCC (location not specified), cloacogenic (n=2) and adeno (n=2) carcinomas	NR	14.9	Log-rank analysis, multivariable Cox regression	Referral from public hospital, HIV status, T stage, RT technique, RT duration, RT delay	Good

**16**	Epidermal Growth Factor Receptor Expression As Prognostic Marker in Patients With Anal Carcinoma Treated With Concurrent Chemoradiation Therapy [Fraunholz et al. (2013)]	2013	Multi-centre, Europe	Retrospective	103	1989-2011	3D-CRT	SCC, basaloid, cloacogenic (location not specified)	7	44.0	Log-rank analysis, multivariable Cox regression	Age, sex, HIV status, T stage, N stage, grade, EGFR expression	Good
**17**	Leukocytosis and neutrophilia predicts outcome in anal cancer [Schernberg et al. (2017)*]	2017	Single centre, Europe	Retrospective	103	2006-2016	IMRT (53%) and 3D-CRT (47%)	SCC (location not specified)	6	38.7	Log-rank analysis, multivariable Cox regression	Leukocytosis, neutrophilia, anaemia, T stage, N stage, CRT duration	Good
**18**	The ongoing challenge of large anal cancers: prospective long term outcomes of intensity-modulated radiation therapy with concurrent chemotherapy [Hosni et al. (2018)]	2018	Single centre, North America	Retrospective	101	2008-2013	IMRT	SCC of the anal canal, SCC of the anal canal with perianal extension	7	56.5	Univariable Cox regression, multivariable Cox regression	T stage, N stage, sex, age, grade, maximum tumour size, RT interruption	Fair
**19**	The impact of anaemia on treatment outcome in patients with squamous cell carcinoma of anal canal and anal margin [Oblak et al. (2016)]	2016	Single centre, Europe	Retrospective	100	2003-2013	3D-CRT and IMRT	SCC of the anal canal and anal margin	7	52.0	Log-rank analysis, multivariable Cox regression	Pre-treatment Hb, on-treatment Hb, end-of-treatment Hb, performance status, T stage, N stage, stage, histology, tumour site, blood transfusion, overall radiation time, operation	Good

Appendix D. Outcome definitions given in each study, stratified into nine categories. The final stratification yielded three disease activity outcome categories and six survival outcome categories.

#	Outcomes (number of studies reporting outcome)	Outcomes included	Study	Definition
1	**Overall survival (n=17)**	Overall survival	Martin et al. (2020) [21]	Survival times were calculated from start of CRT to the date of respective events or last follow-up. Assessed with death of any cause as the respective event.
		Overall survival	de Bellefon et al. (2020) [22]	Calculated starting from the first day of radiotherapy and defined as follows: death from any cause.
		Overall survival	Rouard et al. (2019) [24]	The time between the first day of RT and the death (all causes). Surviving patients were censored at the date of last follow-up or five years after D1.
		Overall survival	Franco et al. (2018) [25]	Calculated from the date of diagnosis to that of death from any cause or lost at observation.
		Overall survival	Call et al. (2016) [26]	Not defined.
		Overall survival	Balermpas et al. (2017) [27]	Calculated from the beginning of CRT to death for any reasons or to cancer-related death, or the day of the last follow-up.
		Overall survival	Rodel et al. (2018) [28]	Defined from the beginning of CRT to the day of death from any reasons.
		Overall survival	Schernberg et al. (2017) [29]	The time between the diagnosis and the time of death.
		Overall survival	Martin et al. (2019) [30]	Calculated from start of CRT to the date of event or last follow-up. Assessed with death of any cause as the respective event.
		Overall survival	Oehler-Janne et al. (2008) [31]	Calculated from the beginning of RT to the day of death or the date of last follow-up.
		Overall survival	Susko et al. (2020) [32]	The time from last radiation treatment to date of death or last follow-up.
		Overall survival	Cardenas et al. (2017) [33]	Not defined.
		Overall survival	Bitterman et al. (2015) [34]	The time from initiation of CRT to death due to any cause or most recent follow-up.
		Overall survival	Fraunholz et al. (2013) [35]	The time from start of CRT until death resulting from any cause, or the date of last follow-up visit.
		Overall survival	Schernberg et al. (2017)* [36]	Not defined
		Overall survival	Hosni et al. (2018) [37]	Not defined
		Overall survival	Oblak et al. (2016) [38]	The time interval from the beginning of the treatment to the death due to any cause.
2	**Locoregional failure (n=11)**	Locoregional recurrence	Shakir et al. (2020) [15]	All failures at site of primary tumor, within the pelvis or inguinal nodes, with or without distant failure, including both patients who failed to achieve CR at 6 months and those occurring more than 6 months after completion of CRT after initial CR.
		Local failure	Shakir et al. (2020) [15]	Persistence or recurrence at the site of initial primary tumor. The site of failure was determined based on physical examination, imaging, and pathology.
		Regional failure	Shakir et al. (2020) [15]	Persistence or recurrence elsewhere in the pelvis or inguinal nodes at any point. The site of failure was determined based on physical examination, imaging, and pathology.

		Local relapse-free survival	Martin et al. (2020) [21]	Survival times were calculated from start of CRT to the date of respective events or last follow-up. Calculated using non-complete response at first restaging or locoregional recurrence after initial complete response as event.
		Locoregional failure	de Bellefon et al. (2020) [22]	Calculated starting from the first day of radiotherapy and defined as follows: residual disease, local and/or regional recurrences.
		Locoregional recurrence	Rouard et al. (2019) [24]	The time between the first day of RT and the date of first local or regional recurrence.
		Local recurrence	Rouard et al. (2019) [24]	The time between the first day of RT and the date of local recurrence.
		Regional recurrence	Rouard et al. (2019) [24]	The time between the first day of RT and the date of regional recurrence.
		Local control	Call et al. (2016) [26]	Defined as the time to local relapse. *No definition for locoregional failure given. Local and regional failure definitions stated separately only.
		Regional control	Call et al. (2016) [26]	Defined as the time to regional relapse. *No definition for locoregional failure given. Local and regional failure definitions stated separately only.
		Cumulative incidence of locoregional failure	Balermpas et al. (2017) [27]	Calculated from the beginning of CRT to non-complete response at restaging or locoregional tumor detection after initial complete response.
		Cumulative incidence of locoregional failure	Rodel et al. (2018) [28]	The time to non-complete response at restaging or locoregional tumour detection after initial complete response. All time-to-event end points were measured from the start of CRT.
		Locoregional control	Schernberg et al. (2017) [29]	The time between the diagnosis and the time of loco-regional recurrence.
		Locoregional control rate	Martin et al. (2019) [30]	Calculated from start of CRT to the date of event or last follow-up. Calculated using non-complete response at first restaging or locoregional recurrence after initial complete response as event.
		Freedom from local recurrence	Susko et al. (2020) [32]	The time from last radiation treatment to locally recurrent disease or last follow-up.
		Locoregional control	Oblak et al. (2016) [38]	The time interval from the beginning of the treatment to the appearance of local and/or regional progression.
**3**	**Disease-free survival (n=11)**	Disease-free survival	Martin et al. (2020) [21]	Survival times were calculated from start of CRT to the date of respective events or last follow-up. Calculated using the date of diagnosis of locoregional failure, distant metastases, or death of any cause.
		Disease-free survival	de Bellefon et al. (2020) [22]	Calculated starting from the first day of radiotherapy and defined as follows: death from any cause or recurrence.
		Progression-free survival	Brown et al. (2019) [23]	Comprises of locoregional failure (LRF), new distant metastatic disease and death, based on which occurred first.
		Disease-free survival	Rouard et al. (2019) [24]	The time between the first day of RT and the date of local, regional or metastatic recurrence or death, whichever occurred first.
		Progression-free survival	Franco et al. (2018) [25]	The time interval between diagnosis and disease recurrence and/or progression at any site, death or lost at follow-up.
		Disease-free survival	Balermpas et al. (2017) [27]	Measured from the beginning of CRT to the day of locoregional failure or distant recurrence, or death from any cause.
		Progression-free survival	Schernberg et al. (2017) [29]	The time between the diagnosis and the time of recurrence or death.
		Disease-free survival	Martin et al. (2019) [30]	Calculated from start of CRT to the date of event or last follow-up. Calculated using the date of diagnosis of locoregional failure, distant metastases, or death of any cause.
		Disease-free survival	Bitterman et al. (2015) [34]	The time from initiation of CRT to the occurrence of local, regional, or distant recurrence, death, or most recent follow-up.
		Progression-free survival	Schernberg et al. (2017)* [36]	Not defined
		Disease-free survival	Hosni et al. (2018) [37]	Not defined

4	**Distant failure (n=5)**	Distant relapse	Shakir et al. (2020) [15]	Development of disease outside of the pelvis or inguinal nodes independent of locoregional status at any point. Failure within the common iliac nodes was considered distant failure.
		Distant control	Call et al. (2016) [26]	Defined as the time to distant relapse.
		Cumulative incidence of distance metastases	Rodel et al. (2018) [28]	Any occurrence of distant metastasis during CRT, at re-staging, or during follow-up. All time-to-event end points were measured from the start of CRT.
		Freedom from distant metastasis	Susko et al. (2020) [32]	The time from last radiation treatment to distant recurrence of disease or last follow-up.
		Distant metastases control	Schernberg et al. (2017)* [36]	The time between the diagnosis and the time of distant metastasis.
5	**Metastasis-free survival (n=5)**	Distant metastasis-free survival	Martin et al. (2020) [21]	Survival times were calculated from start of CRT to the date of respective events or last follow-up. Calculated using the date of diagnosis of distant metastases or death of any cause as event.
		Metastasis-free survival	de Bellefon et al. (2020) [22]	Calculated starting from the first day of radiotherapy and defined as follows: death or distant relapse.
		Distant metastasis-free survival	Martin et al. (2019) [30]	Calculated from start of CRT to the date of event or last follow-up. Calculated using the date of diagnosis of distant metastases or death of any cause as event.
		Distant metastases-free survival	Fraunholz et al. (2013) [35]	The time from the start of CRT to the diagnosis of distant metastases or to death, or the date of last follow-up visit.
		Distant failure-free survival	Schernberg et al. (2017)* [36]	Not defined
6	**Freedom from disease (n=4)**	Disease-free survival	Shakir et al. (2020) [15]	Event defined as either a failure to achieve CR at 6 months or subsequent relapse (local, regional, or distant).
		Time to failure	Shakir et al. (2020) [15]	Interval from start of CRT to date of detection of recurrence. Last follow-up was considered the last clinic visit or date of death.
		Disease-free survival	Rodel et al. (2018) [28]	Defined from the beginning of CRT to the day of locoregional failure or distant recurrence.
		Time to recurrence	Oehler-Janne et al. (2008) [31]	Calculated from the beginning of RT to the day of recurrence or the date of last follow-up.
		Disease-free survival	Oblak et al. (2016) [38]	The time interval from the beginning of the treatment to the appearance of local and/or regional progression and/or appearance of distant metastases.
7	**Colostomy-free survival (n=4)**	Colostomy-free survival	de Bellefon et al. (2020) [22]	Calculated starting from the first day of radiotherapy and defined as follows: death or definitive colostomy. A colostomy performed before radiotherapy was considered as a failure on the first day of treatment as long as it was not reversed later on.
		Colostomy-free survival	Call et al. (2016) [26]	Defined as the time to the date of a colostomy procedure.
		Colostomy-free survival	Bitterman et al. (2015) [34]	Measured from initiation of CRT to diverting colostomy or salvage abdominoperineal resection (APR), death, or most recent follow-up without surgery.
		Colostomy-free survival	Hosni et al. (2018) [37]	Not defined
8	**Cancer-specific survival (n=3)**	Cancer-specific survival	de Bellefon et al. (2020) [22]	Calculated starting from the first day of radiotherapy and defined as follows: death from SCCAC.
		Cancer-specific survival	Fraunholz et al. (2013) [35]	The time from start of CRT until death resulting from the cancer, or the date of last follow-up visit.
		Disease-specific survival	Oblak et al. (2016) [38]	The time interval from the beginning of the treatment to the death because of cancer.
9	**Local failure-free survival (n=2)**	Local recurrence-free survival	Cardenas et al. (2017) [33]	Not defined.
		Local failure-free survival	Fraunholz et al. (2013) [35]	The time from start of CRT to the first local tumor detection after CRT (ie. noncomplete response or local tumor recurrence after complete response) or to death (if the latter event occurred before a local failure was diagnosed), or the date of last follow-up visit.

Appendix E. All outcomes reported in each study, along with all factors tested in both univariable and multivariable analysis.

#	Study	Outcomes	Factors identified as prognostic using univariable analysis	Factors identified as prognostic using multivariable analysis
**1**	Shakir et al. (2020) [15]	Locoregional recurrence, distant relapse, persistent disease, disease-free survival, overall survival	Sex, performance status, T stage, N stage, RT completion, chemotherapy type	Sex, N stage, RT completion, performance status
**2**	Martin et al. (2020) [21]	Local relapse free survival, distant metastasis-free survival, disease-free survival, overall survival	T stage, N stage, gender, high grade acute organ toxicity	T stage, N stage, gender, high grade acute organ toxicity
**3**	de Bellefon et al. (2020) [22]	Locoregional failure, overall survival, colostomy-free survival, disease-free survival, metastasis-free survival	T stage, AJCC stage, N stage, exclusive RT, lack of MMC, RT breaks	T stage, N stage, AJCC stage, sex, RT breaks, exclusive RT, lack of MMC, residual disease
**4**	Brown et al. (2018) [23]	Progression-free survival	*N/A - No univariable analysis performed.*	T stage, N stage, planned total RT dose, planned total RT fractions, Minimum CT value, GLCM entropy log10- PET, GLCM entropy log2- PET, NGLDM busyness- PET, total SMTV, total TLG
**5**	Rouard et al. (2019) [24]	Overall survival, locoregional recurrence, local recurrence, regional recurrence	Age, immunodepression, definitive RT break, anal tumour boost technique, anal tumour total dose, performance status, active smoking, differentiation, lack of MMC, N stage, external iliac involvement at diagnosis, inguinal involvement at diagnosis, keratinisation, PLNA with NC delineation, involved LN not boosted, internal iliac delineation	Age, immunodepression, performance status, active smoking, external iliac involvement at diagnosis, PLNA with NC delineation
**6**	Franco et al. (2018) [25]	Progression-free survival, overall survival	Sex, N stage, basal haemoglobin levels, response to treatment	Sex, N stage, basal haemoglobin levels, response to treatment
**7**	Call et al. (2016) [26]	Overall survival, local control, regional control, distant control, colostomy-free survival	N stage, T stage	N stage, T stage, RT duration
**8**	Balermpas et al. (2017) [27]	Cumulative incidence of locoregional failure, disease-free survival, overall survival	Age, sex, T stage, N stage, HPV16 load, p16, CD8, PD-1, PD-L1, FOXP3, pCASP-8	Age, sex, HPV16 load, p16, CD8, PD-1, PD-L1, FOXP3, pCASP-8
**9**	Rodel et al. (2018) [28]	Cumulative incidence of locoregional failure, cumulative incidence of distance metastases, disease-free survival, overall survival	Gender, T stage, N stage, HPV16 load, RITA expression	Gender, T stage, N stage, HPV16 load, RITA expression
**10**	Schernberg et al. (2017) [29]	Overall survival, progression-free survival, locoregional control, distant metastases control	Leukocytosis, neutrophilia, anaemia, sex, performance status	Leukocytosis, neutrophilia, anaemia, sex, performance status
**11**	Martin et al. (2019) [30]	Locoregional control rate, disease-free survival, distant metastasis-free survival, overall survival	C reactive Protein to Albumin Ratio (CAR), gender, N stage	C reactive Protein to Albumin Ratio (CAR), N stage

**12**	Oehler-Janne et al. (2008) [31]	Overall survival, time to recurrence	*N/A - No univariable analysis performed.*	N stage, severe acute skin toxicity
**13**	Susko et al. (2020) [32]	Freedom from local recurrence, freedom from distant metastasis, overall survival	T stage, time from diagnosis to RT initiation, RT duration	T stage, time from diagnosis to RT initiation, RT duration
**14**	Cardenas et al. (2017) [33]	Local recurrence–free survival, overall survival	Pretreatment SUVmax, posttreatment SUVmax, ΔSUVmax, 5-FU/MMC chemotherapy, use of IMRT	Posttreatment SUVmax, ΔSUVmax, 5-FU/MMC chemotherapy, use of IMRT
**15**	Bitterman et al. (2015) [34]	Overall survival, disease-free survival, colostomy-free survival	*N/A - No univariable analysis performed.*	T stage, use of IMRT
**16**	Fraunholz et al. (2013) [35]	Local failure-free survival, distant metastases-free survival, cancer-specific survival, overall survival	Sex, T stage, N stage, grade, EGFR expression	Sex, N stage, grade
**17**	Schernberg et al. (2017)* [36]	Overall survival, progression-free survival, locoregional failure-free survival, distant failure-free survival	Leukocytosis, neutrophilia, anaemia, T stage, N stage, CRT duration	Leukocytosis, neutrophilia, anaemia, N stage, CRT duration
**18**	Hosni et al. (2018) [37]	Colostomy-free survival, disease-free survival, colostomy-free survival	*N/A - Univariable analysis performed but no significant prognostic factors identified*	T stage, sex, age, anal canal cancer with perianal extension
**19**	Oblak et al. (2016) [38]	Locoregional control, disease-free survival, disease-specific survival, overall survival	Pretreatment Hb level, mean on-treatment Hb level, end-of-treatment Hb level, performance status, T stage, N stage, overall disease stage, histologic tumour type, tumour site, blood transfusion, overall ratiation time, operation	Pre-treatment Hb level, overall disease stage

Appendix F. Clinical factors identified as prognostic for worse outcomes through univariable and multivariable analysis, stratified by outcome. Where available, factor effects and parameterisation used for analysis are also included.

Univariable analysis						
**Outcome (number of studies reporting outcome)**	**Risk factor**	**Times identified as prognostic**	**Total times tested**	**Factor effect (HR, 95% CI)**	**Note**	**Study**
**Overall survival (n=17)**	Higher N stage	10	16	3.40 (1.59-7.27)	Multiple categories (N0,N1,N2,N3)	Shakir et al. (2020) [15]
				N/A	N0 vs N+	Martin et al. (2020) [21]
				N/A	N0 vs N+	de Bellefon et al. (2020) [22]
				2.11 (1.31-2.90)	N0 vs N+	Franco et al. (2018) [25]
				N/A	Multiple categories (N0,N1,N2,N3)	Call et al. (2016) [26]
				N/A	N0 vs N+	Balermpas et al. (2017) [27]
				N/A	N0 vs N+	Rodel et al. (2018) [28]
				N/A	N0 vs N+	Fraunholz et al. (2013) [35]
				N/A	N0 vs N+	Schernberg et al. (2017)* [36]
				N/A	N0 vs N+	Oblak et al. (2016) [38]
	Higher T stage	9	16	4.15 (1.21-14.25)	Multiple categories (T1,T2,T3,T4)	Shakir et al. (2020) [15]
				N/A	T1-2 vs T3-4	Martin et al. (2020) [21]
				N/A	T1-2 vs T3-4	de Bellefon et al. (2020) [22]
				N/A	T1-2 vs T3-4	Balermpas et al. (2017) [27]
				N/A	T1-2 vs T3-4	Rodel et al. (2018) [28]
				N/A	T1-2 vs T3-4	Fraunholz et al. (2013) [35]
				N/A	T1-2 vs T3-4	Schernberg et al. (2017)* [36]
				3.59 (1.30-9.88)	T1-2 vs T3-4	Hosni et al. (2018) [37]
				N/A	T1-3 vs T4	Oblak et al. (2016) [38]
	Male sex	7	12	2.93 (1.64-5.24)	Female/Male	Shakir et al. (2020) [15]
				N/A	Female/Male	Martin et al. (2020) [21]
				2.23 (1.42-3.05)	Female/Male	Franco et al. (2018) [25]
				N/A	Female/Male	Balermpas et al. (2017) [27]
				N/A	Female/Male	Rodel et al. (2018) [28]
				N/A	Female/Male	Schernberg et al. (2017) [29]
				3.38 (1.09-10.50)	Female/Male	Hosni et al. (2018) [37]
	Worse performance status	3	4	11.61 (2.56-52.75)	Multiple categories (PS0,PS1,PS2,PS3)	Shakir et al. (2020) [15]
				N/A	0 vs 1/2	Schernberg et al. (2017) [29]
				N/A	0 vs 1-3	Oblak et al. (2016) [38]
	Older age	3	4	2.15 (1.16-3.98)	<65 vs ≥65	Rouard et al. (2019) [24]
				N/A	≤59 vs >59	Balermpas et al. (2017) [27]
				1.05 (1.00-1.09)	Continuous	Hosni et al. (2018) [37]
	Incomplete/interrupted RT or breaks	2	2	6.21 (2.98-12.95)	No/Yes	Shakir et al. (2020) [15]
				3.25 (1.15-9.13)	No/Yes	Rouard et al. (2019) [24]

	Longer CRT duration	2	5	N/A	No/Yes	Schernberg et al. (2017)* [36]
				N/A	≤ 1.08 months vs > 1.08 months	Oblak et al. (2016) [38]
	Immunodepression	1	1	3.70 (1.30-10.51)	Yes/No	Rouard et al. (2019) [24]
	External RT	1	2	2.38 (1.05-5.55)	Brachytherapy vs External RT	Rouard et al. (2019) [24]
	Lower anal tumour total dose	1	3	2.04 (1.04-4.00)	≥64Gy vs <64Gy	Rouard et al. (2019) [24]
	No response to treatment	1	1	6.26 (2.73-14.40)	Yes/No	Franco et al. (2018) [25]
	Diagnosis to RT initiation	1	1	1.02 (1.00-1.04)	Continuous	Susko et al. (2020) [32]
	Lack of 5-FU/MMC chemotherapy	1	1	12.5	No/Yes	Cardenas et al. (2017) [33]
	Higher tumour grade	1	5	N/A	G1-2 vs G3	Fraunholz et al. (2013) [35]
	Anal canal cancer with perianal extension	1	1	3.04 (1.10-8.38)	No/Yes	Hosni et al. (2018) [37]
	Larger maximum primary tumor size	1	3	1.16 (1.02-1.32)	Continuous	Hosni et al. (2018) [37]
	Higher AJCC stage	1	1	N/A	I/II vs IIIA/IIIB	Oblak et al. (2016) [38]
	Histologic tumour type	1	2	N/A	Basaloid vs squamous	Oblak et al. (2016) [38]
	Blood transfusion	1	1	N/A	No/Yes	Oblak et al. (2016) [38]
	Operation	1	1	N/A	No/Yes	Oblak et al. (2016) [38]
**Locoregional failure (n=11)**	Higher N stage	7	11	3.05 (1.63-5.73)	Multiple categories (N0,N1,N2,N3)	Shakir et al. (2020) [15]
				N/A	N0 vs N+	Martin et al. (2020) [21]
				N/A	Multiple categories (N0,N1,N2,N3)	Call et al. (2016) [26]
				N/A	N0 vs N+	Balermpas et al. (2017) [27]
				N/A	N0 vs N+	Rodel et al. (2018) [28]
				N/A	N0 vs N+	Martin et al. (2019) [30]
				N/A	N0 vs N+	Oblak et al. (2016) [38]
	Higher T stage	7	11	5.17 (1.55-17.28)	Multiple categories (T1,T2,T3,T4)	Shakir et al. (2020) [15]
				N/A	T1-2 vs T3-4	Martin et al. (2020) [21]
				N/A	T1-2 vs T3-4	Call et al. (2016) [26]
				N/A	T1-2 vs T3-4	Balermpas et al. (2017) [27]
				N/A	T1-2 vs T3-4	Rodel et al. (2018) [28]
				4.43 (1.93-10.16)	Multiple categories (T1,T2,T3,T4)	Susko et al. (2020) [32]
				N/A	T1-3 vs T4	Oblak et al. (2016) [38]
	Male sex	5	9	1.78 (1.09-2.91)	Female/Male	Shakir et al. (2020) [15]
				N/A	Female/Male	Martin et al. (2020) [21]
				N/A	Female/Male	Balermpas et al. (2017) [27]
				N/A	Female/Male	Rodel et al. (2018) [28]
				N/A	Female/Male	Schernberg et al. (2017) [29]
	Worse performance status	4	4	1.90 (1.14-3.19)	Multiple categories (PS0,PS1,PS2,PS3)	Shakir et al. (2020) [15]
				3.01 (1.05-8.66)	No/Yes	Rouard et al. (2019) [24]
				N/A	0 or 1 vs ≥2	Schernberg et al. (2017) [29]
				N/A	0 vs 1-3	Oblak et al. (2016) [38]
	Longer RT duration	2	2	1.05 (1.02-1.08)	Continuous	Susko et al. (2020) [32]
				N/A	≤ 1.08 months vs > 1.08 months	Oblak et al. (2016) [38]
	Incomplete/interrupted RT	1	2	5.29 (2.83-9.90)	No/Yes	Shakir et al. (2020) [15]
	Active smoking	1	1	2.22 (1.07-4.61)	No/Yes	Rouard et al. (2019) [24]
	Differentiation	1	1	4.31 (1.25-14.89)	Poorly/moderately/well differentiated	Rouard et al. (2019) [24]

	Lack of MMC chemotherapy	1	1	2.56 (1.16-5.88)	Yes/No	Rouard et al. (2019) [24]
	Immunodepression	1	1	3.54 (1.06-11.83)	No/Yes	Rouard et al. (2019) [24]
	External RT	1	1	3.57 (1.05-12.5)	Brachytherapy vs External RT	Rouard et al. (2019) [24]
	Higher number of involved LN at diagnosis	1	1	3.69 (1.24-11.04)	<2 vs ≥2	Rouard et al. (2019) [24]
	External iliac involvement at diagnosis	1	1	4.65 (1.55-13.93)	No/Yes	Rouard et al. (2019) [24]
	Inguinal involvement at diagnosis	1	1	3.16 (1.10-9.11)	No/Yes	Rouard et al. (2019) [24]
	No keratinisation	1	1	3.13 (1.03-9.10)	Yes/No	Rouard et al. (2019) [24]
	Higher PLNA with NC delineation	1	1	5.77 (1.29-25.78)	<10 vs ≥10	Rouard et al. (2019) [24]
	Higher number of involved LN not boosted	1	1	3.30 (1.03-10.52)	0 or 1 vs ≥2	Rouard et al. (2019) [24]
	NC Internal iliac delineation	1	1	4.20(1.17-15.08)	Conforming vs NC	Rouard et al. (2019) [24]
	Longer time to RT initiation from diagnosis	1	1	1.05 (1.02-1.08)	Continuous	Susko et al. (2020) [32]
	Higher AJCC stage	1	1	N/A	I/II vs IIIA/IIIB	Oblak et al. (2016) [38]
	Squamous histologic tumour type	1	1	N/A	Basaloid vs squamous	Oblak et al. (2016) [38]
	Operation	1	1	N/A	No/Yes	Oblak et al. (2016) [38]
**Disease-free survival (n=11)**	Male sex	5	8	N/A	Female/Male	Martin et al. (2020) [21]
				N/A	Female/Male	Balermpas et al. (2017) [27]
				N/A	Female/Male	Schernberg et al. (2017) [29]
				N/A	Female/Male	Martin et al. (2019) [30]
				2.33 (1.00-5.46)	Female/Male	Hosni et al. (2018) [37]
	Higher N stage	4	9	N/A	N0 vs N+	Martin et al. (2020) [21]
				N/A	N0 vs N+	de Bellefon et al. (2020) [22]
				N/A	N0 vs N+	Balermpas et al. (2017) [27]
				N/A	N0 vs N+	Martin et al. (2019) [30]
	Higher T stage	4	10	N/A	T1-2 vs T3-4	Martin et al. (2020) [21]
				N/A	T1-2 vs T3-4	de Bellefon et al. (2020) [22]
				N/A	T1-2 vs T3-4	Rodel et al. (2018) [28]
				6.25 (2.70-17.40)	T1-2 vs T3-4	Hosni et al. (2018) [37]
	Worse performance status	1	2	N/A	0 vs 1/2	Schernberg et al. (2017) [29]
	High grade acute organ toxicity	1	1	N/A	No/Yes	Martin et al. (2020) [21]
	Anal canal cancer with perianal extension	1	1	2.92 (1.26-6.75)	No/Yes	Hosni et al. (2018) [37]
	Larger maximum primary tumor size	1	2	1.23 (1.12-1.34)	Continuous	Hosni et al. (2018) [37]
**Distant failure (n=5)**	Male sex	1	3	N/A	Female/Male	Rodel et al. (2018) [28]
	Higher T stage	1	5	N/A	T1-2 vs T3-4	Rodel et al. (2018) [28]
	Higher N stage	1	5	N/A	N0 vs N+	Rodel et al. (2018) [28]
	Worse performance status	1	1	N/A	0 vs 1/2	Schernberg et al. (2017) [29]
**Metastasis-free survival (n=5)**	Higher T stage	5	5	N/A	T1-2 vs T3-4	Martin et al. (2020) [21]
				N/A	T1-2 vs T3-4	de Bellefon et al. (2020) [22]
				N/A	T1-2 vs T3-4	Martin et al. (2019) [30]
				N/A	T1-2 vs T3-4	Fraunholz et al. (2013) [35]
				N/A	T1-2 vs T3-4	Schernberg et al. (2017)* [36]

	Higher N stage	4	5	N/A	N0 vs N+	Martin et al. (2020) [21]
				N/A	N0 vs N+	Martin et al. (2019) [30]
				N/A	N0 vs N+	Fraunholz et al. (2013) [35]
				N/A	N0 vs N+	Schernberg et al. (2017)* [36]
	Male sex	2	4	N/A	Female/Male	Martin et al. (2020) [21]
				N/A	Female/Male	Martin et al. (2019) [30]
	Higher tumour grade	1	3	N/A	In HIV- patients only	Fraunholz et al. (2013) [35]
	Longer CRT duration	1	1	N/A	<50 days vs >50 days	Schernberg et al. (2017)* [36]
**Freedom from disease (n=4)**	Higher N stage	4	4	4.02 (2.25-7.17)	Multiple categories (N0,N1,N2,N3)	Shakir et al. (2020) [15]
				N/A	N0 vs N+	Rodel et al. (2018) [28]
				N/A	N0 vs N+	Oblak et al. (2016) [38]
				N/A	In HIV- patients	Oehler-Janne et al. (2008) [31]
	Male sex	2	3	1.85 (1.18-2.92)	Female/Male	Shakir et al. (2020) [15]
				N/A	Female/Male	Rodel et al. (2018) [28]
	Higher T stage	2	3	4.48 (1.56-12.87)	Multiple categories (T1,T2,T3,T4)	Shakir et al. (2020) [15]
				N/A	T1-3 vs T4	Oblak et al. (2016) [38]
	Worse performance status	1	3	1.94 (1.21-3.12)	Multiple categories (PS0,PS1,PS2,PS3)	Shakir et al. (2020) [15]
	Incomplete/interrupted RT	1	1	4.98 (2.74-9.05)	No/Yes	Shakir et al. (2020) [15]
	Severe acute skin toxicity	1	1	N/A	No/Yes In HIV- patients	Oehler-Janne et al. (2008) [31]
	Higher AJCC stage	1	1	N/A	I/II vs IIIA/IIIB	Oblak et al. (2016) [38]
	Squamous histologic tumour type	1	2	N/A	Basaloid vs squamous	Oblak et al. (2016) [38]
	Longer overall radiation time	1	1	N/A	≤ 1.08 months vs > 1.08 months	Oblak et al. (2016) [38]
	Operation	1	1	N/A	No/Yes	Oblak et al. (2016) [38]
**Colostomy-free survival (n=4)**	Higher T stage	3	4	N/A	T3-4 vs T1-2	de Bellefon et al. (2020) [22]
				N/A	T3-4 vs T1-2	Call et al. (2016) [26]
				3.83 (1.68-8.77)	T3-4 vs T1-2	Hosni et al. (2018) [37]
	Anal canal cancer with perianal extension	1	1	3.47 (1.56-7.74)	No/Yes	Hosni et al. (2018) [37]
	Larger maximum primary tumour size	1	1	1.18 (1.08-1.29)	Continuous	Hosni et al. (2018) [37]
**Cancer-specific survival (n=3)**	Higher T stage	2	3	N/A	T1-2 vs T3-4	Fraunholz et al. (2013) [35]
				N/A	T1-3 vs T4	Oblak et al. (2016) [38]
	Higher N stage	2	3	N/A	N0 vs N+	Fraunholz et al. (2013) [35]
				N/A	N0 vs N+	Oblak et al. (2016) [38]
	Higher tumour grade	1	1	N/A	G1-2 vs G3	Fraunholz et al. (2013) [35]
	Higher AJCC stage	1	3	N/A	I/II vs IIIA/IIIB	Oblak et al. (2016) [38]
	Longer overall radiation time	1	1	N/A	≤ 1.08 months vs > 1.08 months	Oblak et al. (2016) [38]
	Operation	1	1	N/A	No/Yes	Oblak et al. (2016) [38]
**Local failure-free survival (n=2)**	Lack of 5-FU/MMC chemotherapy	1	1	4.76	No/Yes	Cardenas et al. (2017) [33]
	Lack of IMRT radiotherapy	1	1	5.56	No/Yes	Cardenas et al. (2017) [33]
	Male sex	1	1	N/A	Female/Male	Fraunholz et al. (2013) [35]
	Higher T stage	1	2	N/A	T1-2 vs T3-4	Fraunholz et al. (2013) [35]
	Higher N stage	1	2	N/A	N0 vs N+	Fraunholz et al. (2013) [35]
	Higher tumour grade	1	1	N/A	G1-2 vs G3	Fraunholz et al. (2013) [35]

Multivariable analysis					
Outcome (number of studies reporting outcome)	Factor	Times identified as prognostic	Factor effect (HR, 95% CI)	Note	Study
Overall survival (n=17)	Male sex	7	4.00 (2.11-7.56)	Female/Male	Shakir et al. (2020) [15]
			1.92 (1.10-3.45)	Female/Male	Martin et al. (2020) [21]
			3.66 (1.56-8.60)	Female/Male	Franco et al. (2018) [25]
			3.13 (1.47-6.66)	Female/Male	Balermpas et al. (2017) [27]
			3.05 (1.42-6.55)	Female/Male	Rodel et al. (2018) [28]
			4.80 (1.60-14.50)	Female/Male	Schernberg et al. (2017) [29]
			4.50 (1.42-14.27)	Female/Male	Hosni et al. (2018) [37]
	Higher T stage	3	4.91 (2.25-10.72)	T1-2 vs T3-4	de Bellefon et al. (2020) [22]
			2.88 (1.12-7.46)	T1-2 vs T3-4	Bitterman et al. (2015) [34]
			4.98 (1.69-14.72)	T1-2 vs T3-4	Hosni et al. (2018) [37]
	Older age	3	2.43 (1.29-4.60)	<65 vs ≥65	Rouard et al. (2019) [24]
			2.32 (1.13-4.73)	≤59 vs >59	Balermpas et al. (2017) [27]
			1.05 (1.00-1.09	Continuous	Hosni et al. (2018) [37]
	Higher N stage	3	2.25 (1.00-5.17)	N0 vs N+	Franco et al. (2018) [25]
			1.88 (1.16-3.10)	Multiple categories (N0,N1,N2,N3)	Call et al. (2016) [26]
			5.80	N0 vs N+	Schernberg et al. (2017)* [36]
	Higher AJCC stage	2	2.82 (1.22-6.53)	I/II/III vs IV	de Bellefon et al. (2020) [22]
			2.23 (1.17-4.26)	I/II vs IIIA/IIIB	Oblak et al. (2016) [38]
	Worse performance status	1	10.71 (1.94-58.95)	Multiple categories (PS0,PS1,PS2,PS3)	Shakir et al. (2020) [15]
	Incomplete/interrupted RT	1	4.22 (1.78-10.00)	No/Yes	Shakir et al. (2020) [15]
	Exclusive RT	1	3.38 (1.29-10.72)	No/Yes	de Bellefon et al. (2020) [22]
	Lack of MMC	1	1.88 (0.92-3.85)	No/Yes	de Bellefon et al. (2020) [22]
	Immunodepression	1	5.05 (1.72-14.80)	No/Yes	Rouard et al. (2019) [24]
	No response to treatment	1	6.96 (2.96–16.50)	Yes/No	Franco et al. (2018) [25]
	Longer diagnosis to RT initiation	1	1.02 (1.00-1.05)	Continuous	Susko et al. (2020) [32]
	Lack of 5-FU/MMC chemotherapy	1	9.09	No/Yes	Cardenas et al. (2017) [33]
	Lack of IMRT radiotherapy	1	4.00 (1.30-12.5)	No/Yes	Bitterman et al. (2015) [34]
Locoregional failure (n=11)	Male sex	4	2.08 (1.24-3.48)	Female/Male	Shakir et al. (2020) [15]
			2.22 (1.16-4.38)	Female/Male	Martin et al. (2020) [21]
			2.56 (1.04-6.25)	Female/Male	Balermpas et al. (2017) [27]
			3.40 (1.30-9.40)	Female/Male	Schernberg et al. (2017) [29]
	Higher N stage	3	2.23 (1.13-4.39)	Multiple categories (N0,N1,N2,N3)	Shakir et al. (2020) [15]
			3.00 (1.55-5.81)	N0 vs N+	Martin et al. (2020) [21]
			3.58 (1.25-10.26)	N0 vs N+	Martin et al. (2019) [30]
	Incomplete/interrupted RT or breaks	2	4.96 (2.40-10.27)	No/Yes	Shakir et al. (2020) [15]

			2.47 (1.15-5.30)	No/Yes	de Bellefon et al. (2020) [22]
	Worse performance status	2	3.82 (1.31-11.09)	<2 vs ≥2	Rouard et al. (2019) [24]
			5.50 (2.20-14.00)	0 vs 1/2	Schernberg et al. (2017) [29]
	Exclusive RT	1	3.41 (1.21-9.57)	No/Yes	de Bellefon et al. (2020) [22]
	Lack of MMC	1	3.11 (1.28-7.56)	No/Yes	de Bellefon et al. (2020) [22]
	Active smoking	1	2.31 (1.11-4.82)	No/Yes	Rouard et al. (2019) [24]
	Immunodepression	1	7.25 (1.54-34.20)	No/Yes	Rouard et al. (2019) [24]
	External iliac involvement at diagnosis	1	7.89 (2.54-24.56)	No/Yes	Rouard et al. (2019) [24]
	Higher PLNA with NC delineation	1	9.09 (1.96-42.15)	<10 vs ≥10	Rouard et al. (2019) [24]
	Higher T stage	1	4.37 (1.83-10.47)	Multiple categories (T1,T2,T3,T4)	Susko et al. (2020) [32]
	Longer time to RT initiation from diagnosis	1	1.06 (1.03-1.010)	Continuous	Susko et al. (2020) [32]
**Disease-free survival (n=11)**	Male sex	4	2.13 (1.19-3.85)	Female/Male	Martin et al. (2020) [21]
			2.27 (2.38-4.35)	Female/Male	Balermpas et al. (2017) [27]
			3.60 (1.50-8.60)	Female/Male	Schernberg et al. (2017) [29]
			2.46 (1.04-5.73)	Female/Male	Hosni et al. (2018) [37]
	Higher T stage	3	2.57 (1.42-4.66)	Categorical	de Bellefon et al. (2020) [22]
			7.02 (2.76-17.83)	T1-2 vs T3-4	Hosni et al. (2018) [37]
			NA	Multiple categories (T1,T2,T3,T4), variable weighting reported (-0.011)	Brown et al. (2019) [23]
	Higher N stage	2	3.06 (1.70-5.49)	N0 vs N+	Martin et al. (2020) [21]
			NA	Multiple categories (N0,N1,N2,N3), variable weighting reported (-0.019)	Brown et al. (2019) [23]
	Lower planned total RT dose	1	NA	Continuous, variable weighting reported (0.007)	Brown et al. (2019) [23]
	Fewer planned total RT fractions	1	NA	Continuous, variable weighting reported (0.012)	Brown et al. (2019) [23]
	High grade acute organ toxicity	1	2.13 (1.20-3.70)	No/Yes	Martin et al. (2020) [21]
	Higher AJCC stage	1	2.23 (0.99-5.01)	I/II/III vs IV	de Bellefon et al. (2020) [22]
	Worse performance status	1	4.90 (2.10-11.50)	0 vs 1/2	Schernberg et al. (2017) [29]
	Longer CRT duration	1	33.33	<50 days vs >50 days	Schernberg et al. (2017)* [36]
**Distant failure (n=5)**	Male sex	1	3.83 (1.20-12.27)	Female/Male	Rodel et al. (2018) [28]
	Higher T stage	1	4.24 (1.43-12.57)	T1-2 vs T3-4	Rodel et al. (2018) [28]
**Metastasis-free survival (n=5)**	Male sex	2	4.08 (1.63-10.19)	Female/Male	Martin et al. (2020) [21]
			3.87 (1.08-13.84)	Female/Male	Fraunholz et al. (2013) [35]
	Higher T stage	2	3.54 (1.52-8.23)	T1-2 vs T3-4	Martin et al. (2020) [21]
			2.61 (1.45-4.70)	T1-2 vs T3-4	de Bellefon et al. (2020) [22]
	Higher N stage	2	2.41 (1.0405.62)	N0 vs N+	Martin et al. (2020) [21]
			4.49 (1.20-16.80)	N0 vs N+	Martin et al. (2019) [30]
	Higher AJCC stage	1	3.05 (1.41-6.62)	I/II/III vs IV	de Bellefon et al. (2020) [22]
	Higher tumour grade	1	5.88 (1.72-20.00)	G1-2 vs G3	Fraunholz et al. (2013) [35]
**Freedom from disease (n=4)**	Male sex	2	2.16 (1.34-3.48)	Female/Male	Shakir et al. (2020) [15]
			2.16 (1.09-4.26)	Female/Male	Rodel et al. (2018) [28]
	Higher N stage	1	2.73 (1.43-5.21)	Multiple categories (N0,N1,N2,N3)	Shakir et al. (2020) [15]
	Incomplete/interrupted RT	1	4.50 (2.26-8.97)	No/Yes	Shakir et al. (2020) [15]

**Colostomy-free survival (n=4)**	Higher T stage	3	4.10 (2.23-7.52)	T1-2 vs T3-4	de Bellefon et al. (2020) [22]
			4.00 (1.03-17.09)	T1-2 vs T3-4	Call et al. (2016) [26]
			3.65 (1.59-8.37)	T1-2 vs T3-4	Hosni et al. (2018) [37]
	Male sex	1	1.90 (1.10-3.10)	Female/Male	de Bellefon et al. (2020) [22]
	Residual disease	1	7.78 (3.41-17.77)	No/Yes	de Bellefon et al. (2020) [22]
	Exclusive RT	1	3.03 (1.39-6.57)	No/Yes	de Bellefon et al. (2020) [22]
	Anal canal cancer with perianal extension	1	3.17 (1.42-7.09)	No/Yes	Hosni et al. (2018) [37]
**Cancer-specific survival (n=3)**	Male sex	1	4.13 (1.24-13.63)	Female/Male	Fraunholz et al. (2013) [35]
	Higher N stage	1	6.25 (1.51-25.00)	N0 vs N+	Fraunholz et al. (2013) [35]
	Higher AJCC stage	1	3.52 (1.38-9.03)	I/II vs IIIA/IIIB	Oblak et al. (2016) [38]

Appendix G. Biomarkers identified as prognostic for worse outcomes through univariable and multivariable analysis, stratified by outcome. Where available, factor effects and parameterisation used for analysis are also included.

Univariable analysis						
**Outcome (number of studies reporting outcome)**	**Factor**	**Times identified as prognostic**	**Total times tested**	**Factor effect (HR, 95% CI)**	**Note**	**Study**
**Overall survival (n=17)**	Lower HPV16 load	2	3	N/A	>/≤ median	Balermpas et al. (2017) [27]
				N/A	>/≤ median	Rodel et al. (2018) [28]
	Neutrophilia	2	2	N/A	Absent vs present (neutrophils >7G/L)	Schernberg et al. (2017) [29]
				N/A	Absent vs present (neutrophils >7500/mm3)	Schernberg et al. (2017)* [36]
	Anaemia	2	2	N/A	Absent vs present (hemoglobin count < 13.0 g/dL)	Schernberg et al. (2017) [29]
				N/A	Absent vs present (hemoglobin count < 13.0 g/dL)	Schernberg et al. (2017)* [36]
	Lower basal heamoglobin levels	1	1	2.00 (1.20-3.33)	Continuous	Franco et al. (2018) [25]
	Lower CD8 expression	1	1	N/A	>/≤ median	Balermpas et al. (2017) [27]
	Lower PD-1 expression	1	1	N/A	>/≤ median	Balermpas et al. (2017) [27]
	Lower RITA expression	1	1	N/A	>/≤ WS6	Rodel et al. (2018) [28]
	Leukocytosis	1	2	N/A	Present (leukocytes >10G/L) vs absent	Schernberg et al. (2017) [29]
	High C reactive protein to albumin ratio	1	1	N/A	≤/> 0.117	Martin et al. (2019) [30]
	Lower pre-treatment heamoglobin levels	1	1	N/A	> 120 g/L vs ≤ 120 g/L	Oblak et al. (2016) [38]
	Lower mean on-treatment heamoglobin levels	1	1	N/A	> 120 g/L vs ≤ 120 g/L	Oblak et al. (2016) [38]
	Lower end-of-treatment heamoglobin levels	1	1	N/A	> 120 g/L vs ≤ 120 g/L	Oblak et al. (2016) [38]
**Locoregional failure (n=11)**	Lower HPV16 load	2	3	N/A	>/≤ median	Balermpas et al. (2017) [27]
				N/A	>/≤ median	Rodel et al. (2018) [28]
	Lower p16 expression	1	1	N/A	>/≤ median	Balermpas et al. (2017) [27]
	Lower CD8 expression	1	1	N/A	>/≤ median	Balermpas et al. (2017) [27]
	Lower PD-1 expression	1	1	N/A	>/≤ median	Balermpas et al. (2017) [27]
	Lower PD-L1 expression	1	1	N/A	>/≤ median	Balermpas et al. (2017) [27]
	Weaker FOXP3 phosporylation	1	1	N/A	>/≤ median	Balermpas et al. (2017) [27]
	Weaker pCasp-8 phosporylation	1	1	N/A	>/≤ median	Balermpas et al. (2017) [27]
	Lower RITA expression	1	1	N/A	>/≤ WS6	Rodel et al. (2018) [28]
	Leukocytosis	1	1	N/A	Absent vs present (leukocytes >10G/L)	Schernberg et al. (2017) [29]
	Neutrophilia	1	1	N/A	Absent vs present (neutrophils >7G/L)	Schernberg et al. (2017) [29]
	Anaemia	1	1	N/A	Absent vs present (hemoglobin count < 13.0 g/dL)	Schernberg et al. (2017) [29]
	High C reactive protein to albumin ratio	1	1	N/A	≤/> 0.117	Martin et al. (2019) [30]
	Lower pre-treatment heamoglobin levels	1	1	N/A	> 120 g/L vs ≤ 120 g/L	Oblak et al. (2016) [38]
	Lower mean on-treatment heamoglobin levels	1	1	N/A	> 120 g/L vs ≤ 120 g/L	Oblak et al. (2016) [38]
	Lower end-of-treatment heamoglobin levels	1	1	N/A	> 120 g/L vs ≤ 120 g/L	Oblak et al. (2016) [38]

**Disease-free survival (n=11)**	Leukocytosis	2	2	N/A	Absent vs present (leukocytes >10G/L)	Schernberg et al. (2017) [29]
				N/A	Absent vs present (leukocytes >10000/mm3)	Schernberg et al. (2017)* [36]
	Neutrophilia	2	2	N/A	Absent vs present (neutrophils >7G/L)	Schernberg et al. (2017) [29]
				N/A	Absent vs present (neutrophils >7500/mm3)	Schernberg et al. (2017)* [36]
	Lower CD8 expression	1	1	N/A	>/≤ median	Balermpas et al. (2017) [27]
	Lower PD-1 expression	1	1	N/A	>/≤ median	Balermpas et al. (2017) [27]
	Weaker FOXP3 phosporylation	1	1	N/A	>/≤ median	Balermpas et al. (2017) [27]
	Weaker pCasp-8 phosporylation	1	1	N/A	>/≤ median	Balermpas et al. (2017) [27]
	Lower HPV16 load	1	2	N/A	>/≤ median	Balermpas et al. (2017) [27]
	Anaemia	1	2	N/A	Absent vs present (hemoglobin count < 13.0 g/dL)	Schernberg et al. (2017) [29]
	High C reactive protein to albumin ratio	1	1	N/A	≤/> 0.117	Martin et al. (2019) [30]
**Distant failure (n=5)**	Lower HPV16 load	1	1	N/A	>/≤ median	Rodel et al. (2018) [28]
	Lower RITA expression	1	1	N/A	>/≤ WS6	Rodel et al. (2018) [28]
	Leukocytosis	1	1	N/A	Absent vs present (leukocytes >10G/L)	Schernberg et al. (2017) [29]
	Neutrophilia	1	1	N/A	Absent vs present (neutrophils >7G/L)	Schernberg et al. (2017) [29]
**Metastasis-free survival (n=5)**	High C reactive protein to albumin ratio	1	1	N/A	≤/> 0.117	Martin et al. (2019) [30]
	Leukocytosis	1	1	N/A	Absent vs present (leukocytes >10000/mm3)	Schernberg et al. (2017)* [36]
	Neutrophilia	1	1	N/A	Absent vs present (neutrophils >7500/mm3)	Schernberg et al. (2017)* [36]
	Anaemia	1	1	N/A	Absent vs present (hemoglobin count < 13.0 g/dL)	Schernberg et al. (2017)* [36]
**Freedom from disease (n=4)**	Lower HPV16 load	1	1	N/A	>/≤ median	Rodel et al. (2018) [28]
	Lower RITA expression	1	1	N/A	>/≤ WS6	Rodel et al. (2018) [28]
	Lower pre-treatment heamoglobin levels	1	1	N/A	> 120 g/L vs ≤ 120 g/L	Oblak et al. (2016) [38]
	Lower end-of-treatment heamoglobin levels	1	1	N/A	> 120 g/L vs ≤ 120 g/L	Oblak et al. (2016) [38]
**Cancer-specific survival (n=3)**	EGFR expression	1	1	N/A	Intermediate/Intense vs Absent/Weak	Fraunholz et al. (2013) [35]
	Lower pre-treatment heamoglobin levels	1	1	N/A	> 120 g/L vs ≤ 120 g/L	Oblak et al. (2016) [38]
	Lower end-of-treatment heamoglobin levels	1	1	N/A	> 120 g/L vs ≤ 120 g/L	Oblak et al. (2016) [38]
**Local failure-free survival (n=2)**	Leukocytosis	1	1	N/A	Absent vs present (leukocytes >10000/mm3)	Schernberg et al. (2017)* [36]
	Neutrophilia	1	1	N/A	Absent vs present (neutrophils >7500/mm3)	Schernberg et al. (2017)* [36]
**Multivariable analysis**						
**Outcome (number of studies reporting outcome)**	**Factor**	**Times identified as prognostic**	**Total times tested**	**Factor effect (HR, 95% CI)**	**Note**	**Study**
**Overall survival (n=17)**	Leukocytosis	2		4.60 (1.40-14.90)	Absent vs present (leukocytes >10G/L)	Schernberg et al. (2017) [29]
				19.90	Absent vs present (leukocytes >10000/mm3)	Schernberg et al. (2017)* [36]
	Neutrophilia	2		4.40 (1.30-14.80)	Absent vs present (neutrophils >7G/L)	Schernberg et al. (2017) [29]
				22.70	Absent vs present (neutrophils >7500/mm3)	Schernberg et al. (2017)* [36]
	Lower basal heamoglobin levels	1		1.89 (1.15-3.03)	Continuous	Franco et al. (2018) [25]
	Lower HPV16 load	1		2.27 (1.05-5.00)	>/≤ median	Balermpas et al. (2017) [27]
	Lower RITA expression	1		3.19 (1.29-7.86)	>/≤ WS6	Rodel et al. (2018) [28]

	High C reactive protein to albumin ratio	1	4.47 (1.53-13.03)	≤/> 0.117	Martin et al. (2019) [30]
	Anaemia	1	5.40	Absent vs present (hemoglobin count < 13.0 g/dL)	Schernberg et al. (2017)* [36]
	Lower pre-treatment heamoglobin levels	1	2.38 (1.08-5.26)	> 120 g/L vs ≤ 120 g/L	Oblak et al. (2016) [38]
**Locoregional failure (n=11)**	Lower HPV16 load	2	3.57 (1.29-10)	>/≤ median	Balermpas et al. (2017) [27]
			4.51 (1.15-13.46)	>/≤ median	Rodel et al. (2018) [28]
	Lower p16 expression	1	3.13 (1.30-7.14)	>/≤ median	Balermpas et al. (2017) [27]
	Lower CD8 expression	1	4.00 (1.20-14.29)	>/≤ median	Balermpas et al. (2017) [27]
	Lower PD-1 expression	1	3.45 (1.39-8.33)	>/≤ median	Balermpas et al. (2017) [27]
	Lower PD-L1 expression	1	3.70 (1.11-12.5)	>/≤ median	Balermpas et al. (2017) [27]
	Lower RITA expression	1	4.35 (1.45-13.02)	>/≤ WS6	Rodel et al. (2018) [28]
	Leukocytosis	1	4.50 (1.30-15.60)	Absent vs present (leukocytes >10G/L)	Schernberg et al. (2017) [29]
	Neutrophilia	1	3.60 (1.20-11.60)	Absent vs present (neutrophils >7G/L)	Schernberg et al. (2017) [29]
	Anaemia	1	4.10 (1.30-12.40)	Absent vs present (hemoglobin count < 13.0 g/dL)	Schernberg et al. (2017) [29]
**Disease-free survival (n=11)**	Leukocytosis	2	7.10 (2.50-20.20)	Absent vs present (leukocytes >10G/L)	Schernberg et al. (2017) [29]
			6.90	Absent vs present (leukocytes >10000/mm3)	Schernberg et al. (2017)* [36]
	Neutrophilia	2	5.00 (1.70-14.50)	Absent vs present (neutrophils >7G/L)	Schernberg et al. (2017) [29]
			7.60	Absent vs present (neutrophils >7500/mm3)	Schernberg et al. (2017)* [36]
	Anaemia	2	5.30 (1.90-14.70)	Absent vs present (hemoglobin count < 13.0 g/dL)	Schernberg et al. (2017) [29]
			2.50	Absent vs present (hemoglobin count < 13.0 g/dL)	Schernberg et al. (2017)* [36]
	Lower CD8 expression	1	2.38 (1.15-5.00)	>/≤ median	Balermpas et al. (2017) [27]
	Lower PD-1 expression	1	2.17 (1.16-4.00)	>/≤ median	Balermpas et al. (2017) [27]
	Weaker FOXP3 phosporylation	1	1.85 (1.00-3.45)	>/≤ median	Balermpas et al. (2017) [27]
	Weaker pCasp-8 phosporylation	1	2.04 (1.06-3.84)	>/≤ median	Balermpas et al. (2017) [27]
	Lower HPV16 load	1	2.50 (1.27-5.00)	>/≤ median	Balermpas et al. (2017) [27]
**Distant failure (n=5)**	Leukocytosis	1	4.00 (1.60-10.30)	Absent vs present (leukocytes >10G/L)	Schernberg et al. (2017) [29]
	Neutrophilia	1	3.30 (1.20-9.10)	Absent vs present (neutrophils >7G/L)	Schernberg et al. (2017) [29]
**Metastasis-free survival (n=5)**	Leukocytosis	1	N/A	Absent vs present (leukocytes >10000/mm3)	Schernberg et al. (2017)* [36]
	Neutrophilia	1	N/A	Absent vs present (neutrophils >7500/mm3)	Schernberg et al. (2017)* [36]
	Anaemia	1	N/A	Absent vs present (hemoglobin count < 13.0 g/dL)	Schernberg et al. (2017)* [36]
**Freedom from disease (n=4)**	Lower HPV16 load	1	2.28 (1.08-4.79)	>/≤ median	Rodel et al. (2018) [28]
	Lower RITA expression	1	2.19 (1.07-4.47)	>/≤ WS6	Rodel et al. (2018) [28]
**Local failure-free survival (n=2)**	Leukocytosis	1	N/A	Absent vs present (leukocytes >10000/mm3)	Schernberg et al. (2017)* [36]
	Neutrophilia	1	N/A	Absent vs present (neutrophils >7500/mm3)	Schernberg et al. (2017)* [36]

Appendix H. Imaging factors identified as prognostic for worse outcomes through univariable and multivariable analysis, stratified by outcome. Where available, factor effects are also included.

Univariable analysis					
**Outcome (number of studies reporting outcome)**	**Factor**	**Times identified as prognostic**	**Total times tested**	**Factor effect (HR)**	**Study**
**Overall survival (n=17)**	Higher posttreatment SUVmax	1	1	3.23	Cardenas et al. (2017) [33]
	Smaller ΔSUVmax	1	1	4.35	Cardenas et al. (2017) [33]
**Local failure-free survival (n=2)**	Lower pretreatment SUVmax	1	1	3.57	Cardenas et al. (2017) [33]
	Higher posttreatment SUVmax	1	1	4.35	Cardenas et al. (2017) [33]

Multivariable analysis				
**Outcome (number of studies reporting outcome)**	**Factor**	**Times identified as prognostic**	**Factor effect**	**Study**
**Overall survival (n=17)**	Higher posttreatment SUVmax	1	2.77	Cardenas et al. (2017) [33]
	Smaller ΔSUVmax	1	3.33	Cardenas et al. (2017) [33]
**Distant failure (n=5)**	Minimum CT value	1	N/A	Brown et al. (2019) [23]
	GLCM entropy log10- PET	1	N/A	Brown et al. (2019) [23]
	GLCM entropy log2- PET	1	N/A	Brown et al. (2019) [23]
	NGLDM busyness- PET	1	N/A	Brown et al. (2019) [23]
	Total SMTV	1	N/A	Brown et al. (2019) [23]
	Total TLG	1	N/A	Brown et al. (2019) [23]
**Local failure-free survival (n=1)**	Higher posttreatment SUVmax	1	5.88	Cardenas et al. (2017) [33]
